# Measuring transparency in intelligent robots

**DOI:** 10.1038/s41598-025-29685-w

**Published:** 2025-12-12

**Authors:** Georgios Angelopoulos, Dimitri Lacroix, Ricarda Wullenkord, Alessandra Rossi, Silvia Rossi, Friederike Eyssel

**Affiliations:** 1 Interdepartmental Center for Advances in Robotic Surgery - ICAROS, University of Naples Federico II, 80131 Naples, Italy; 2 Center for Cognitive Interaction Technology - CITEC, Bielefeld University, 33619 Bielefeld, Germany

**Keywords:** Transparency, Human-Robot Interaction, Psychometrics, Scale, Human behaviour, Engineering

## Abstract

As robots become increasingly integrated into our daily lives, the need to make them transparent has never been more critical. Yet, despite its importance in human-robot interaction, a standardized measure of robot transparency has been missing until now. This paper addresses this gap by presenting the first comprehensive scale to measure perceived transparency in robotic systems, available in English, German, and Italian languages. Our approach conceptualizes transparency as a multidimensional construct, encompassing explainability, legibility, predictability, and meta-understanding. The proposed scale was a product of a rigorous three-stage process involving 1223 participants. Firstly, we generated the items, secondly, we conducted an exploratory factor analysis, and thirdly, a confirmatory factor analysis served to validate the factor structure of the newly developed TOROS scale. The final scale encompasses 26 items and comprises three factors: *Illegibility*, *Explainability*, and *Predictability*. TOROS demonstrates high cross-linguistic reliability, inter-factor correlation, model fit, internal consistency, and convergent validity across the three cross-national samples. This empirically validated tool enables the assessment of robot transparency and contributes to the theoretical understanding of this complex construct. By offering a standardized measure, we facilitate consistent and comparable research in human-robot interaction in which TOROS can serve as a benchmark.

## Introduction

Humans rely on perception, interpretation, and anticipation to interact effectively with their environment, and the same applies when interacting with robots^1^. When a robot behaves in unexpected or unclear ways, users may need to pause, hesitate, or intervene, which can disrupt smooth collaboration, reduce efficiency, or even compromise safety^2^. Ensuring that robot actions are understandable and predictable is therefore crucial for effective interactions. This characteristic is often discussed under the term transparency, which has become a central concern in robotics and artificial intelligence^3^.

Transparency is explicitly recognized as an important issue in governance frameworks. The Principles of Robotics issued by the UK’s Engineering and Physical Sciences Research Council state that “Robots are manufactured artefacts. They should not be designed in a deceptive way to exploit vulnerable users; instead, their machine nature should be transparent”^4^. At the European level, the Artificial Intelligence Act (EU AI Act) further highlights transparency as a legal requirement. Article 13 stipulates that “AI systems shall be designed and developed in such a way to ensure that their operation is sufficiently transparent to enable providers and users to reasonably understand the system’s functioning”^5^. These frameworks demonstrate that transparency is not simply a desirable design principle but a mandated condition for the deployment of AI and robotic systems in society^6,7^.

Despite this recognition, a clear and commonly accepted definition of transparency in human-robot interaction (HRI) is still missing. Some authors define transparency in the sense of predictability, such as “Transparency is essentially the opposite of unpredictability” ^8^, p. 193. Accordingly, if a robot provides cues that support its users in anticipating its next actions and states, the robot should be deemed more predictable and, thus, transparent. In contrast, other definitions of transparency emphasize the aspect of legibility. Wortham et al.^9^, p. 274 defined transparency as “... the extent to which the robot’s ability, intent, and situational constraints are understood by users”. According to Kim and Hinds^10^, p. 81, the notion of explainability is also intertwined with transparency. They propose “Transparency is the robot offering explanations of its actions”. These explanations can be provided before an action is performed, during an action, or after performing an action^11,12^. Recent works, however, combine the aforementioned aspects, suggesting that transparency in HRI should encompass what a robot is doing (legibility), why it is doing that (explainability), and what it will do next (predictability)^13–15^. Therefore, we take a multifaceted initial approach to transparency, integrating elements of explainability, legibility, and predictability.

Transparency of robot behavior is essential for several reasons: For end-users, it regulates expectations, fosters trust, acceptance, and improves user experience^16–19^. For developers, it aids processes associated with design, debugging, and evaluation by making robot behavior easier to inspect and adjust^3,20^. However, transparency is a double-edged sword: While transparency can enhance trust and acceptance, it may also result in negative consequences, such as overtrust^21^ or information overload. For instance, when explanations provided by the robot are too extensive^22^. Transparency is distinct from related constructs like trust or acceptance, in that it concerns whether the system’s functioning can be understood, not whether it is deemed reliable or benevolent (trust), nor enjoyable or useful (acceptance). As such, transparency deserves to be measured as a construct on its own.

Neverthless, measuring transparency remains problematic. Schött et al.^23^ have highlighted the absence of consensus regarding the reliability and validity of metrics employed to assess the transparency of a robot’s behavior. In addition, Claure et al.^24^ and Bartneck et al.^25^ underscored the critical need for developing a standardized measurement framework to assess transparency. When measuring transparency, straightforward questions such as “Is the robot transparent?” or “Is the robot predictable?” might seem adequate at first glance^26,27^. However, such face-valid, single-item measures fail to comprehensively capture all facets of a complex construct like transparency, leading to inconsistent and unreliable data^28^. The lack of consensus regarding the measurement of transparency in HRI research suggests that it may be understood differently from one individual to another. Similarly, lay people may perceive transparency differently from HRI researchers and practitioners, who distinguish explainability, legibility, and predictability as dimensions of transparency. For instance, legibility and predictability are not easily distinguishable in terms of human understanding. In fact, human cognition is deeply based on predictive processes to understand the environment and prepare individuals for actions^29,30^. It is then possible that people, when understanding something, have the feeling that it is predictable as well^31^. The development of a validated scale is therefore essential for methodological rigor and for the theoretical understanding of transparency.

**Fig. 1:**
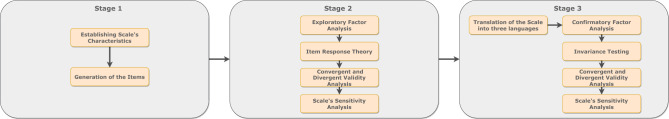
The three stages of the scale development.

The present paper addresses exactly this gap. To the best of our knowledge, it proposes the first measurement instrument to assess the perceived transparency of a robot’s behavior. We present the validation process, which follows the state-of-the-art for scale creation^28,32^. This process comprises three distinct stages, as shown in Fig. 1, each integral to ensure the robustness and validity of the resulting final scale. These stages consist of (1) Initial item generation to formulate a set of items, (2) Exploratory Factor Analysis (EFA) to discern a factor arrangement, and (3) a Confirmatory Factor Analysis (CFA) to validate the scale’s factor structure. Factor analyzes are a crucial step to explore and validate a measurement, as they narrow down a large number of variables (e.g., the items of a scale) to a smaller number of factors (e.g., the expected dimensions of a scale), using patterns of correlation between variables. Whereas EFAs are used to extract factors by grouping correlated variables, CFAs served to test how an already assumed factor structure fit a set of observed variables^33^. The research protocol was approved by Bielefeld University’s Ethics Review Board (application number 2023-349). Participants provided informed consent and the research protocol was in accordance with the Declaration of Helsinki.

## Stage 1

Stage 1 of the scale development involved an examination of existing definitions and measures to allow the creation of the first version of a scale assessing a robot’s perceived transparency. A literature review suggested that a robot’s behavior requires three characteristics to be transparent: explainability, legibility, and predictability^13–15^. However, transparency also emerges from how these dimensions help the human perceiver to build a mental model of the robot’s functioning^34,35^. We refer to this fourth aspect as meta-understanding. With these four characteristics established, we generated items that effectively capture each aspect of transparency. Here, we were guided by the methodological recommendation to generate three to five times the number of items intended for a resulting measurement instrument, with at least four items per dimension^32^. Consequently, 64 items in total were initially developed and distributed evenly across the four dimensions. Moreover, since no validated transparency scale is available in the literature yet, the first version of the scale was designed so items from previous studies that explicitly measured specific aspects of transparency (e.g., *“I find the robot’s behavior easy to understand”*) were incorporated, as explained in recent HRI and Human-Computer Interaction (HCI) literature^15,23,36^. Each item was formulated to carefully match the theoretical notions associated with explainability, legibility, predictability, and meta-understanding. This way, we made sure that the items were relevant and sufficient to fully capture each dimension.

Furthermore, in Stage 1, we deliberately created multiple items with similar content or phrasing. The rationale behind this strategy was two-fold. First, it allowed for a more comprehensive coverage of the construct’s content domain, ensuring that various nuances and aspects of each dimension are captured. Second, it provides the opportunity to empirically evaluate which items perform best in terms of psychometric properties during subsequent phases of scale refinement. By including redundant items at this stage (e.g., *“The robot’s behavior is predictable”*, *“The robot’s actions are unpredictable”*), we increase the likelihood of identifying the most robust and discriminating items for the final scale, potentially improving its overall reliability and validity^37^.

In addition, to capture the different ways in which individuals might perceive and describe their experiences with robots, a mix of personal and nonpersonal statements was developed. Personal statements (e.g., *“I can [...]”, “I find [...]”, “I feel [...]”*) refer to direct expressions of the participant’s experiences and feelings. Nonpersonal statements (e.g., *“The robot’s behavior”, “The robot’s explanation [...]”, “The robot’s actions”*) provide an assessment of the robot’s characteristics. For each expected dimension of transparency, we complemented these regular items (i.e., statements phrased in the direction of the targeted constructs, also known as positively worded items)^38,39^ with three reverse-worded items (asking for agreement with statements phrased in the opposite direction of the targeted construct)^39–41^, each for personal and non-personal statements (e.g., *“I find it difficult [...]”, “The robot does not [...]”*). This was done to counter-response the acquiescence bias (i.e., tendency of respondents to use agreement with statement as the default answer to spare effort), careless responding, and to increase the reliability of responses^40^. Responses are measured using a 7-point Likert scale, ranging from *“Strongly Disagree” (1)* to *“Strongly Agree” (7)*. Each answer point is fully labelled, which increases the understandability of the items, reduces cognitive load of respondents, and limitates the risk of misresponse to reverse-worded items^38^. 7-point Likert scales offer an optimal balance between ease of use, adjustment to memory span, and accuracy^42^. In addition, as 7-point Likert scales include a midpoint, they allow to identify answers that are neutral (i.e., lacking strong positive or negative evaluations) or ambivalent (i.e., evaluations that are both strongly positive and negative), which can be valuable leads for further research^43–45^. Even-numbered response formats (e.g., 4- or 6-point scales) were not considered because they prevent participants from expressing neutrality or ambivalence by constraining them to make a choice (i.e., “agree” or “disagree”). Doing so, even-numbered response formats can lead to answers that do not reflect actual evaluations of respondents^38,42,46^, and can elicit negative reactions from ambivalent participants^38,47^. However, prior research has advocated for the use of even-numbered response formats because midpoints in odd-numbered response formats are deemed a threat to reliability^42,46^. Therefore, and despite inconsistent evidence for such an effect^42,46^, it was taken into account when assessing the reliability of the scale at the next Stages. The initial 64 items can be found in Table S1 of the Supplementary Information.

## Stage 2

Following item generation, Stage 2 of the scale development and validation process featured an empirical experimental study. This enabled an exploratory factor analysis to examine the underlying structure of the scale. Such analysis served to confirm the hypothesized four-factor model of perceived transparency. EFA was used because it is instrumental in assessing the scale’s factorial structure and facilitates item reduction by identifying items that strongly contribute to each factor. At this stage, an assessment of the scale’s item difficulty and discrimination parameters was also included. Additionally, Stage 2 served to evaluate the scale’s convergent and divergent validity through controlled manipulations of the primary dimensions of transparency (explainability, legibility, and predictability), as explained in previous works^13–15^, with image vignettes (controlled visual scenarios). This process was crucial in order to confirm whether changes in these dimensions correspond to variations in perceived transparency. That way, we can provide evidence for the scale’s sensitivity and construct validity.

Before Experiment 1, we conducted a pretest to identify a hypothetical everyday life scenario featuring HRI that would effectively discriminate transparency. Thereby, we maximized the efficiency of our experimental manipulation in testing the scale’s sensitivity and effectiveness for measuring transparency. Based on the pretest’s findings, we selected a scenario where a robot is heading towards a charging station to refill its battery. The results of the pretest can be found in the Supplementary Information.

Experiment 1 employed a $2 \times 2 \times 2$ between-subject design which manipulated the explainability, legibility, and predictability of a robot’s behavior as either low or high, using eight image vignettes, selected based on the results of the pretest. Experiment 1 was implemented on Qualtrics. Participants were presented with the purpose and procedure of the experiment. Those who gave their informed consent were randomly assigned to one of the eight between-subject conditions resulting from the manipulation of three variables related to transparency. After viewing the scenario, participants were required to self-report the following dependent variables: Perceived transparency with 64 items developed during Stage 1 (Item Generation), trust towards robots with 20 items from the Multi-Dimensional Measure of Trust (MDMT)^48^, and acceptance of robots using seven subscales from a toolkit based on the Unified Theory of Acceptance and Use of Technology (UTAUT), suggested for HRI research^49^. The order of the items within each scale was randomized. This was done to avoid a potential order effect induced by item presentation^50^, and to vary the alternance of regular and reverse-worded items^40^. Additionally, demographic information (i.e., age, gender, education, self-assessed English language proficiency) was collected, and prior experience with robots was assessed using a scale based on^51^. Finally, two attention checks were included, one at the beginning and one at the end of the study. Only complete datasets from participants over 18 years of age and with a self-declared English proficiency at the A2 level (Elementary) and above were included. Data from people failing both attention checks were excluded. The data collection was planned to conclude after obtaining complete datasets from 320 participants. This sample size was strategically chosen to meet the requirement of having at least 5 participants per item for factor analysis^52^, considering the transparency scale consisted of 64 items. Additionally, the sample size met the recommended threshold of 300 participants for robust factor analysis^33^. Participants for Experiment 1 were recruited via Prolific, and they were reimbursed with £1.50 for participating. The pre-registration for Experiment 1 is available at https://aspredicted.org/NNP_HFM.

### Sample

For Experiment 1, we recruited 371 participants. Following the pre-registered exclusion criteria, 49 participants were removed from the sample; 13 did not complete the study, and 36 were excluded because they did not pass both attention checks. Thus, the final sample comprised $N=322$ participants, slightly exceeding our pre-registered target by two participants. The demographic breakdown, as detailed in Table S3 of the Supplementary Information, was as follows: 182 females, 135 males, two identifying as diverse, two who preferred not to disclose their gender, and one gender-fluid participant. The participants’ age range was between 18 and 71 years ($M=29.820$, $SD=9.340$).

### Results

An EFA using *SPSS 28.0* was used to assess the factorial validity of the 64-item scale. This analysis utilized Principal Axis Factoring (PAF) with an oblique (direct-oblimin) rotation, which allows intercorrelations among factors^53,54^. This approach was accompanied by evaluations of univariate statistics, the Kaiser-Meyer Olkin (KMO) measure of sampling adequacy, Bartlett’s test of sphericity, eigenvalues, and the Scree plot.

**Fig. 2:**
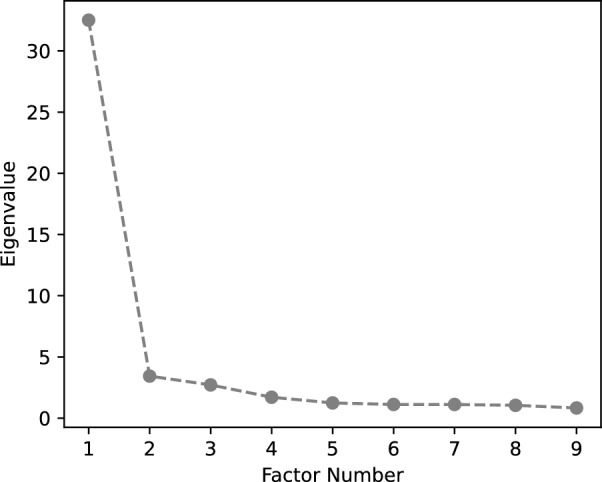
Scree plot illustrating the eigenvalues of extracted factors of the scale in Experiment 1.

The KMO for the sample data was .976. Consequently, the data appeared suitable for an EFA^55,56^. Bartlett’s test further validated the suitability of this statistical method, producing a $\chi ^2 = 18084.169$, $\text {df} =2016$, $p< 0.001$. The Scree plot, in Fig. 2, revealed a clear breakpoint after two factors, suggesting one major factor. However, the curve flattened after five factors, suggesting that the optimal number of factor to retain was four to five^33^. Eigenvalues above 1.0 suggested seven factors. At this stage of the scale development, we opted for a five-factor model (instead of one to four) to avoid discarding potentially meaningful factors before further examination of the items. In addition, the five-factor model was preferred over a seven-factor model to avoid a risk of model overfit. The five factors we have retained accounted for $64.154\%$ of the variance. This result demonstrated the strong explanatory power of the scale.

Initially, all 64 items were retained for the initial EFA. The results, detailing the factor loadings, are depicted in Tables S5 and S6 of the Supplementary Information. Subsequent iterations of the EFA were conducted using the same settings, with item reduction guided by specific criteria to ensure a robust factor structure. These criteria involved removing items with loadings below 0.400 to maintain a strong factor representation and enhance interpretability^57^. Additionally, items loaded on multiple factors with a loading difference of less than 0.100 were excluded to minimize ambiguity in item association^57^. Furthermore, factors containing fewer than three items were removed to ensure reliable and meaningful factor solutions^28,58^.

Initial item removals included five rounds of EFA, in which low factor loadings, multiple high factor loadings, and factor loadings on underrepresented factors were taken into account as exclusion criteria. The final EFA resulted in a refined 31-item scale structured into three distinct factors. The revised factor model demonstrates robust factor loadings. The detailed factor loadings for this definitive structure are presented in Tables S7 and S8 of the Supplementary Information.

Following the iterations of EFA, an inter-item correlation analysis was conducted to minimize redundancy within each factor. Items with excessively high correlations (greater than 0.700 with more than one item) were removed to ensure the distinctiveness of each item within the factors. This approach confirmed that the remaining items clearly and distinctly represented the underlying constructs without undue overlap. At first, the inter-item correlations for Factor 1 were analyzed, with correlation coefficients ranging from 0.511 to 0.793 with a mean of $M= 0.680$. Table S9 of the Supplementary Information shows that four items had correlation coefficients higher than 0.700 with more than one variable, leading to their removal. Table S10 of the Supplementary Information shows the final inter-item correlation matrix of Factor 1 ranging from 0.511 to 0.728 with a mean of $M=0.642$. Afterward, we analyzed Factor 2 (as shown in Table S11 of the Supplementary Information), in which the correlation coefficients ranged from 0.479 to 0.740 with $M=0.607$. One item had correlation coefficients greater than 0.700 with more than one item and was removed. A new analysis was conducted (depicted in Table S12 of the Supplementary Information), showing that the correlation coefficients ranged from 0.479 to 0.690 with a mean of $M=0.598$. Finally, as depicted in Table S13 of the Supplementary Information, the values of the inter-item correlation for Factor 3 ranged from 0.490 to 0.683 with a mean of $M=0.572$. No items were removed, leading to a 26-item scale.

To validate the robustness and appropriateness of the factor structure of the 26-item scale, we checked sampling adequacy, factorability, internal consistency, and convergent validity. The KMO reaffirmed the suitability of the data for EFA with a value of .968. Bartlett’s test supported the factorability of the correlation matrix, yielding a $\chi ^2 = 6217$, $\text {df} =325$, $p<0.001$. The new factor loadings for both pattern and structure matrices were examined to confirm the appropriateness of the items within each factor. Internal consistency was assessed using Cronbach’s Alpha ($\alpha$) and McDonald’s Omega ($\omega$). All factors demonstrated high internal consistency, with Factor 1 recording values of 0.930 for both Cronbach’s Alpha and McDonald’s Omega, Factor 2 showing a Cronbach’s Alpha of 0.926 and McDonald’s Omega of 0.927, and Factor 3 similar to Factor 1 with values of 0.930 for both metrics. The high internal consistency of each factor suggested that the scale is reliable, especially when accounting for a potential negative impact of a 7-point format on it^46^. Composite Reliability (CR) and Average Variance Extracted (AVE) were calculated to evaluate the factors’ convergent validity. The CR values for Factor 1, Factor 2, and Factor 3 were satisfactory at 0.800, 0.865, 0.735, respectively. Regarding the AVE, Factor 1, Factor 2, and Factor 3 recorded values of 0.438, 0.481, and 0.463, respectively. Despite the AVE values being marginally below the ideal threshold of 0.5, the high CR values justify proceeding with the utilization of the scale ^59^. The comprehensive results from these evaluations are presented in Table 1.

**Table 1:** The scale after the removal of items with low loadings and high inter-item correlation in Experiment 1.

Item	Factor pattern loadings			Factor structure loadings	Internal reliability		Convergent validity	
	Factor 1	Factor 2	Factor 3		$\alpha$	$\omega$	CR	AVE
The robot’s overall functioning is a mystery to me.	$-$0.**797**	$-$0.008	0.032	$-$0.683	0.930	0.930	0.800	0.438
It is hard to make sense of the robot’s general functioning.	$-$0.**770**	$-$0.032	0.058	$-$0.656				
It is difficult to get a clear picture of the robot’s overall operations.	$-$0.**706**	$-$0.101	0.021	$-$0.690				
I am confused about the robot’s general objectives.	$-$0.**692**	$-$0.144	0.020	$-$0.715				
I am unsure what the robot does.	$-$0.**688**	$-$0.098	$-$0.038	$-$0.724				
I cannot comprehend the robot’s inner processes.	$-$0.**673**	0.004	$-$0.056	$-$0.641				
I cannot explain the robot’s behavior.	$-$0.**671**	0.135	$-$0.284	$-$0.729				
It is impossible to know what the robot does.	$-$0.**638**	$-$0.049	$-$0.124	$-$0.715				
It is clear to me what the robot does.	**0.467**	0.347	0.106	0.799				
I have a clear understanding of how the robot operates in general.	**0.426**	0.320	0.067	0.739				
I feel like the robot’s explanations are useful.	0.024	**0.832**	$-$0.039	0.688	0.926	0.927	0.865	0.481
The robot explains complex tasks in a way that is easy to understand.	0.119	**0.753**	$-$0.024	0.718				
The robot provides detailed explanations of its actions.	0.008	**0.708**	0.084	0.678				
The robot provides clear explanations for its actions.	0.067	**0.657**	0.163	0.757				
The robot’s explanations for its actions are straightforward0.	0.049	**0.654**	0.223	0.792				
I feel informed about the robot’s activities.	0.251	**0.625**	0.044	0.787				
The robot conveys its overall state effectively.	0.096	**0.600**	0.110	0.688				
It is easy for me to foresee the robot’s future actions.	$-$0.043	$-$0.011	**0.829**	0.685	0.930	0.930	0.735	0.463
The robot’s behavior is predictable.	$-$0.034	0.090	**0.785**	0.739				
I feel confident in predicting the robot’s next moves.	0.057	0.064	**0.732**	0.751				
It is easy to anticipate what will follow the robot’s behavior.	0.057	0.038	**0.723**	0.721				
It is difficult for me to tell what the robot will do next.	$-$0.318	0.168	$-$0.**666**	$-$0.728				
The robot’s next steps are clear to me.	0.049	0.209	**0.656**	0.798				
The robot’s actions are obvious.	$-$0.018	0.266	**0.585**	0.725				
The robot provides cues that help predict its next actions.	$-$0.082	0.336	**0.554**	0.700				
The robot’s behavior does not help predict what it will do next.	$-$0.277	0.076	$-$0.**536**	$-$0.654				

**Fig. 3:**
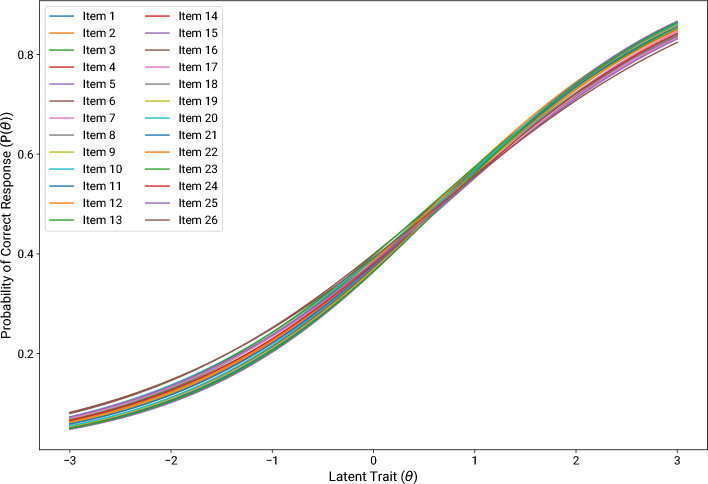
The Item Characteristic Curve exhibits a sigmoid shape.

Moreover, to further evaluate the 26-item scale, we employed Item Response Theory (IRT). We did so to analyze item difficulty and dicrimination^60^, as outlined in Table S14 of the Supplementary Information. IRT is a statistical approach that models the relationship between an individual’s response to an item and their level of the underlying construct being measured, known as the latent trait^32,60,61^. To do so, IRT conceptually distinguishes a respondents’ abilities or attitudes that determine their response to an item from the characteristics of the item, assuming both determine an observed score^60,61^. The IRT model results are presented in Fig. 3 using an Item Characteristic Curve (ICC) for all 26 items. Such ICC featured a sigmoid shape, indicating a good fit between the model and the empirical data ^60^. Specifically, the sigmoid pattern suggests that as the latent trait increases, the probability of a correct response also increases systematically, reflecting an effective measurement scale. For item difficulty, values ranged from 0.580 to 0.710 ($M=0.660$, $SD=0.040$), which is within the acceptable range (i.e., 0.200–0.900) and slightly above the optimal range (i.e., 0.500–0.600, with some authors arguing for 0.500–0.750)^62–64^. As higher values imply lower item difficulty, the scale can be deemed moderately difficult^62–64^. Regarding item discrimination, the range was between 0.660 and 0.810 ($M=0.745$, $SD=0.045$), with values above 0.350–0.600 representing excellent levels of item discrimination^64,65^. Both item difficulty and item discrimination confirm that the items are effective in differentiating between individuals. These results discard a common concern in psychometrics regarding the potentially negative effect of reverse-worded items on the reliability and understandability of a scale^39,66^. Indeed, neither Factor 1 (8 reverse-worded items out of 10) nor Factor 3 (2 reverse-worded items out of 9) are noticeably worse (or better) in terms of reliability, difficulty or discrimination than Factor 2 (no reverse-worded items). It must be noted that the higher number of reverse-worded items in Factor 1 compared to the other factors can be due to an artefact called a method factor. Indeed, regular and reverse-worded items tend to load on different factors in factor analyses, even when they are supposed to measure the same construct^39,41,66^. The presence of such an artefact in the three-factor structure of the scale is examined at the next stage.

At the end of the item removal procedure and scale evaluation, the 26-item scale was composed of three factors. Factor 1 encompassed ten items (two regular, eight reverse-worded) that were assumed to capture perceived legibility (e.g.,“It is impossible to know what the robot does”) and meta-understanding (e.g., “I cannot comprehend the robot’s inner processes”). Overall Factor 1 pertains to a perceived difficulty to understand a robot’s behavior, hindering the possibility to infer its functioning, goals, or underlying processes. Factor 2 comprised seven regular items that were supposed to capture perceived explainability of the robot (e.g., “The robot provides detailed explanations of its actions”). A notable exception is “The robot conveys its overall state effectively”, that was supposed to measure meta-understanding. However, this item probably loaded on items pertaining to explainability because “conveys” implied the ability of the robot to communicate about a “state” that guided its behavior. Finally, Factor 3 included nine items (seven regular, two reverse-worded) that were assumed to capture perceived predictability, with one supposed to measure perceived legibility (i.e., “The robot’s actions are obvious”). Regarding the latter, it is possible that a robot’s actions were deemed “obvious” when they seemed to foreshadow the subsequent ones. These results are consistent with the three-factor approach of transparency: Legibility (Factor 1), Exainability (Factor 2), Predictability (Factor 3)^13–15^. However, Factor 1 mostly included reverse-worded items, and thus tapped more into the polar opposite of legibility: Illegibility. In addition, illegibility hinted more at how a robot’s actions fail to infer internal states, such as goals or functioning, rather than how the actions themselves are understandable. Hence the presence of items hypothetically related to both legibility and meta-understanding. We decided to keep the factors unlabelled until Experiment 2 confirmed these results.

**Fig. 4:**
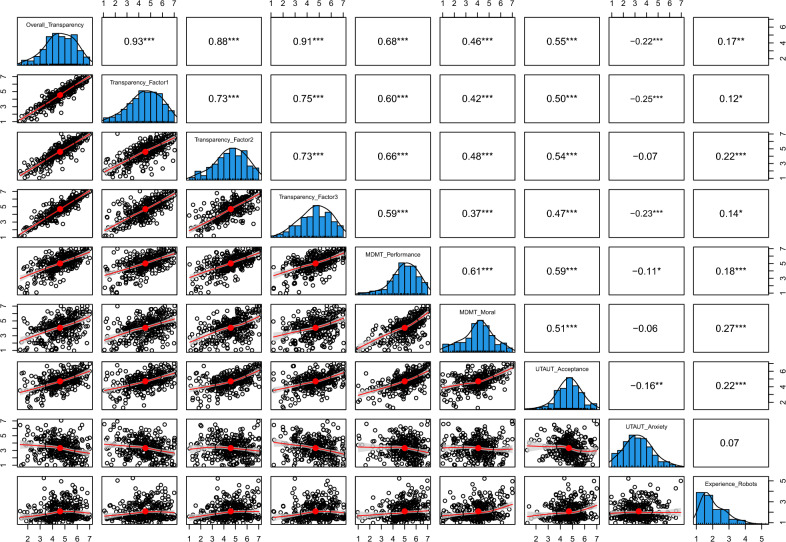
Correlation matrices between the dependent and control variables of Experiment 1.

Prior to assessing the sensitivity of the 26-item scale to an experimental manipulation of transparency of a robot’s behavior, we examined the reliability of the other questionnaires (i.e., MDMT, UTAUT, and experience with robots). Cronbach’s alpha ($>0.7$) confirmed their internal consistency, as demonstrated in Table S15 of the Supplementary Information. Afterward, we conducted a series of 2x2x2 factorial ANCOVAs. A first set of ANCOVAs was conducted to verify that our measurement of perceived transparency of robots’ behavior is sensitive to a manipulation of the dimensions of transparency identified by the available literature. Therefore, manipulated levels of explainability, legibility, and predictability (each set at low versus high) were used as the independent variables while utilizing the means for each factor as well as the mean of all items from the 26-item scale as the dependent variables. A second set of ANCOVAs was dedicated to confirm that transparency of a robot positively influences acceptance and trust towards it. Thus, levels of explainability, legibility, and predictability were used as independent variables, with performance and moral trust (MDMT), acceptance, and anxiety (UTAUT) as dependent variables. In cases where experience with robots was significantly correlated with the tested dependent variable (see Fig. 4), it was included as a covariate in the ANCOVA. Table 2 shows the results of all the ANCOVAs.

We found a significant main effect of manipulated explainability, legibility, and predictability on transparency calculated using the average of all the items. However, no interaction effect between manipulated explainability, legibility, and predictability was identified. Similarly, we obtained significant main effects of manipulated explainability, legibility, and predictability on Factor 1, Factor 2, and Factor 3. However, no interaction effect between manipulated explainability, legibility, and predictability on Factor 1, Factor 2, or Factor 3 was observed. Prior experience with robots was a significant covariate for average perceived transparency, Factor 1, Factor 2, and Factor 3. These results confirmed the sensitivity of our measurement of perceived transparency to an experimental manipulation of the transparency of a robot’s behavior.

Regarding trust towards the robot, we found significant main effects of manipulated explainability and legibility on performance (see Table S16 of the Supplementary Information) and moral trust towards the robot (see Table S17 of the Supplementary Information). However, although the main effect of manipulated predictability on performance trust was significant, we did not find a significant effect of manipulated predictability on moral trust towards the robot. No significant interaction effect between manipulated explainability, legibility, and predictability on performance trust or moral trust was discovered. Prior experience with robots was a significant covariate for performance trust and moral trust towards the robot. Finally, we observed a significant main effect of manipulated explainability, legibility, and predictability on acceptance of the robot (see Table S18 of the Supplementary Information). Prior experience with robots was a significant covariate for acceptance. No interaction effect between manipulated explainability, legibility, and predictability on acceptance was identified. No significant effect of the independent variables on anxiety was found (see Table S19 of the Supplementary Information). These results provided support for the positive effect of transparency of a robot’s behavior on acceptance and trust towards robots.

**Table 2:** Results of the 3-way ANOVA with the manipulation of explainability, legibility, and predictability of the robot’s behavior as independent variables in Experiment 1.

Parameter	df	SS	MS	*F* value	*p* value	$\eta p^2$	95% CI
Dependent variable: Overall Score of the Perceived Transparency							
Explainability condition	1	74.10	74.13	70.53	< .001***	0.18	[0.12, 1.00]
Legibility condition	1	9.20	9.23	8.78	0.003**	0.03	[0.01, 1.00]
Predictability condition	1	27.70	27.67	26.33	< 0.001***	0.08	[0.04, 1.00]
Prior experience with robots	1	17.60	17.64	16.78	< 0.001***	0.05	[0.02, 1.00]
Explainability: Legibility	1	0.70	0.70	0.67	0.415	< 0.01	[0.00, 1.00]
Explainability: Predictability	1	0.30	0.29	0.28	0.599	< 0.01	[0.00, 1.00]
Legibility: Predictability	1	1.90	1.90	1.803	0.180	0.01	[0.00, 1.00]
Explainability: Legibility: Predictability	2	0.60	0.62	0.59	0.444	< 0.01	[0.00, 1.00]
Residuals	313	329.0	1.05				
Dependent variable: Factor 1							
Explainability condition	1	67.50	67.50	46.55	< 0.001***	0.13	[0.08, 1.00]
Legibility condition	1	13.30	13.35	9.21	0.003**	0.03	[0.01, 1.00]
Predictability condition	1	31.80	31.85	21.96	< 0.001***	0.07	[0.03, 1.00]
Prior experience with robots	1	12.50	12.48	8.60	0.004**	0.03	[0.01, 1.00]
Explainability: Legibility	1	0.90	0.94	0.65	0.421	< 0.01	[0.00, 1.00]
Explainability: Predictability	1	0.10	0.07	0.05	0.823	< 0.01	[0.00, 1.00]
Legibility: Predictability	1	2.80	2.83	1.95	0.163	0.01	[0.00, 1.00]
Explainability: Legibility: Predictability	2	1.60	1.58	1.09	0.298	< 0.01	[0.00, 1.00]
Residuals	313	453.90	1.45				
Dependent variable: Factor 2							
Explainability condition	1	116.50	116.45	91.57	< 0.001***	0.23	[0.16, 1.00]
Legibility condition	1	6.60	6.57	5.17	0.024*	0.02	[0.00, 1.00]
Predictability condition	1	17.30	17.32	13.62	< .001***	0.04	[0.01, 1.00]
Prior experience with robots	1	32.60	32.58	25.61	< .001***	0.08	[0.04, 1.00]
Explainability: Legibility	1	1.70	1.73	1.36	0.244	< 0.01	[0.00, 1.00]
Explainability: Predictability	1	0.10	0.12	0.10	0.756	< 0.01	[0.00, 1.00]
Legibility: Predictability	1	1.00	1.00	0.78	0.377	< 0.01	[0.00, 1.00]
Explainability: Legibility: Predictability	2	0.10	0.06	0.05	0.824	< 0.01	[0.00, 1.00]
Residuals	313	398.10	1.27				
Dependent variable: Factor 3							
Explainability condition	1	54.00	54.04	41.52	< 0.001***	0.12	[0.07, 1.00]
Legibility condition	1	7.40	7.42	5.70	0.018*	0.02	[0.00, 1.00]
Predictability condition	1	32.40	32.37	24.87	< 0.001***	0.07	[0.03, 1.00]
Prior experience with robots	1	14.20	14.20	10.91	0.001**	0.03	[0.01, 1.00]
Explainability: Legibility	1	0.10	0.10	0.08	0.781	< 0.01	[0.00, 1.00]
Explainability: Predictability	1	2.50	2.52	1.94	0.165	0.01	[0.00, 1.00]
Legibility: Predictability	1	1.80	1.77	1.36	0.244	< 0.01	[0.00, 1.00]
Explainability: Legibility: Predictability	2	0.50	0.46	0.36	0.551	< 0.01	[0.00, 1.00]
Residuals	313	407.40	1.30				

df Degrees of freedom, *SS* Sum of Squares, *MS* Mean Squares.

*p< 0.05; **p< 0.01; ***p< 0.001.

## Stage 3

Following Stage 2, which concluded with a 26-item scale, we proceeded to Stage 3 of the scale development process. In this stage, we designed Experiment 2 to validate the factor structure. The first step involved translating the 26-item scale into German and Italian, employing a forward and backward translation process by two independent translators^67^. This was essential for testing the scale’s properties across languages and for ensuring cross-linguistic reliability and validity. Additionally, the effectiveness of the scale was assessed by measuring participant responses under conditions of low versus high transparency with video vignettes. These vignettes were based on the scenarios of Stage 2, and the video format was meant to have the transparency of the robot assessed in more ecological settings (i.e., after observing a real robot in action). Additionally, Stage 2 included a CFA to validate the factor structure of the 26-item scale identified in the previous Stage. As a result, Stage 3 was critical in demonstrating the scale’s capacity to discriminate between different levels of perceived transparency, demonstrating its practical applicability and psychometric consistency across diverse cultural contexts, as in previous studies^68^.

Experiment 2 was designed as a 2 (low and high transparency conditions) $\times 3$ (3 languages) between-subject experiment that served to manipulate the transparency of a robot’s behavior via video vignettes featuring the robot Pepper (Softbank Robotics). Given that explainability, legibility, and predictability all had a significant influence on each factor of the scale in the previous stage, for Stage 2 we used two conditions to manipulate the overall transparency. Moreover, Experiment 2 was conducted in three languages (English, German, and Italian). Participants were presented with the purpose and procedure of the experiment. Those who gave informed consent were randomly assigned to one of the two transparency conditions, and the corresponding video was displayed. To ensure that participants watched the entire video featuring the robot behaving high vs. low in transparency at least once, the “Continue” button appeared only after approximately 5 seconds. Following the video, participants completed the resulting refined scale from Stage 2, followed by the MDMT scale^48^ and the subscales from the UTAUT toolkit^49^. Translated versions of these scales, prepared by language experts, were used for German and Italian participants. The order of the items for each scale was randomized. Additionally, demographic information was collected (i.e., participants’ age, gender, education, self-assessed English language proficiency) and prior experience with robots was assessed using a scale based on^51^. Finally, two attention checks were included at the study’s beginning and end, and one memory check was included after the video’s projection. Only complete datasets from participants over 18 years of age and with a self-declared language level (English, German, or Italian, depending on the language condition) above B2 level (Upper Intermediate) were included. Data from people failing the attention checks or the memory check were excluded. Following existing recommendations^33^, the data collection was planned to conclude after obtaining complete datasets from 300 participants per sub-sample, resulting in a total number of 900 complete data sets. The Study 2 pre-registration details are available at https://aspredicted.org/JMC_3B8.

### Sample

We recruited 927 participants using Prolific. Following our pre-registered exclusion criteria, 26 participants were disqualified; 17 failed to complete the study, 1 was excluded for not passing the video attention check, and 8 were removed for insufficient language proficiency. The final sample comprised $N=901$ participants. As detailed in Table S20 of the Supplementary Information, the demographics were as follows: 427 females, 452 males, 14 identifying as diverse, and 8 who preferred not to disclose their gender. The age range of participants was between 18 and 71 years ($M=37.607$, $SD=12.215$), and their experience with robots was rated below average ($M=1.930$, $SD=1.160$). The average duration of participation was recorded at 9.1 minutes, and each participant were compensated with £1.50 for their participation in the study.

### Results

A Confirmatory Factor Analysis using the *lavaan* package in R was employed to verify the factor structure of the 26-item scale. The CFA results for the English, German, and Italian versions of the scale indicated a good model fit for the English and Italian versions, with Comparative Fit Index (CFI) values of 0.930 and 0.943, respectively, and Tucker-Lewis index (TLI) values of 0.924 and 0.937. The Root Mean Square Error of Approximation (RMSEA) values were 0.073 for English and 0.067 for Italian, both within acceptable ranges. The Standardized Root Mean Square Residual (SRMR) values were 0.053 and 0.045, respectively, indicating a good fit. The German version showed weaker fit indices with a CFI of 0.890, TLI of 0.879, RMSEA of 0.093, and SRMR of 0.062, suggesting a less optimal fit than the other versions. Despite this, all factor loadings were significant across all three language versions, indicating that the items loaded well onto their respective factors. Table 3 shows the specific analysis results.

Three alternative Confirmatory Factor analyses were conducted. First, we ran a two-factor CFA where one factor contained all regular items whereas the other factor contained all the reverse worded items. It was conducted to assess the possibility that reverse-worded items in the scale created a method factor. Indeed, reverse-worded items can create an artefact in a factor extraction process, which results in the separation of regular and reverse-worded items supposed to measure a single construct in two factors. The factor containing reverse-worded items is referred to as a method factor^39^. The second alternative CFA assumed a single factor to determine whether the scale can be used to calculate a valid transparency score in lieu of the three subscores, each characterizing a dimension of transparency. For both the two-factor and one-factor CFA, most fit indices did not reach acceptable thresholds, with some of them being marginally acceptable. The third alternative CFA was based on our initial assumption according to which transparency encompasses legibility, explainability, predictability, and meta-understanding. The fit indices from this four-factor model ranged from marginally acceptable to excellent. However, apart from the Goodness of Fit Index of the four-factor model in German language, all fit indices indicated a slightly lower fit than the three-factor model. The comparative fit indices of these CFA for every version of the scale can be consulted on Tables S21-23. Taken together, the CFAs revealed that the three-factor solution had the best fit indices in each tested language and that Factor 1 was likely not a method factor.

**Table 3:** Comparative Fit Indices for English, German, and Italian Models of Experiment 2.

Standards	English	German	Italian	Acceptable	Excellent
Minimum fit function chi-square ($\chi ^2$)	774.253	1067.055	694.216	–	–
Degrees of freedom (*df*)	296	296	296		
$\chi ^2/df$	2.620	3.600	2.350	$<5.00$	$<3.00$
GFI	0.828	0.756	0.841	$>0.80$	$>0.90$
RMSEA	0.073	0.093	0.067	$<0.08$	$<0.06$
AGFI	0.797	0.711	0.811	$>0.80$	$>0.90$
NFI	0.892	0.854	0.905	$>0.85$	$>0.90$
CFI	0.930	0.890	0.943	$>0.90$	$>0.95$
TLI	0.924	0.879	0.937	$>0.90$	$>0.95$
IFI	0.930	0.900	0.940	$>0.90$	$>0.95$
SRMR	0.053	0.062	0.045	$<0.08$	$<0.05$

Following the individual CFAs, measurement invariance testing was done to assess the comparability of the scale across the three language versions. Results indicated configural and metric invariance, but not scalar or residual invariance. This suggests that while the construct and factor loadings are comparable across English, German, and Italian, there are differences in item intercepts and residual variances. Specifically, the configural invariance model, which tests if the basic structure is similar across groups, showed a good fit with a CFI of 0.910, TLI of 0.902, and RMSEA of 0.078, indicating that the factor structure is consistent across languages. The metric invariance model, which constrains factor loadings to be equal across groups, also showed acceptable fit (CFI of 0.906, TLI of 0.907, and RMSEA of 0.082), though the chi-squared difference test was significant, $\Delta \chi ^2 = 120.47$, $p < 0.001$). The scalar invariance model, which additionally constrains item intercepts to be equal across groups, showed a significant deterioration in fit compared to the metric model, $\Delta \chi ^2 = 283.93$, $p < 0.001$, although the overall fit indices remained similar (CFI = 0.906, TLI = 0.907, RMSEA = 0.082). This suggests that while the overall structure and factor loadings are comparable, there may be differences in how participants from different language groups interpret or respond to specific items on the scale. The residual invariance model, which further constrains residual variances to be equal across groups, likewise showed a significant decrease in fit, $\Delta \chi ^2 = 226.99$, $p < 0.001$, with a slight drop in CFI to 0.898. This indicates that the unexplained variance in item responses may differ across language groups. Table 4 shows the results of the invariance testing.

**Table 4:** Invariance Testing Results for Configural, Metric, Scalar, and Residual Models in Experiment 2.

Model	$\chi ^2$	df	$\Delta \chi ^2$	$\Delta$df	CFI	TLI	RMSEA	SRMR
Configural	2535.5	888			0.910	0.902	0.078	0.071
Metric	2656.0	934	120.47*	46	0.906	0.907	0.082	0.071
Scalar	2939.9	980	283.93*	46	0.906	0.907	0.082	0.071
Residual	3166.9	1032	226.99*	52	0.898	0.903	0.083	0.070

*df* Degrees of freedom; $\Delta \chi ^2$ Change in Chi-Square; $\Delta$df Change in Degrees of Freedom

*p< 0.05; **p< 0.01; ***p< 0.001;

After examining scale invariance, it was crucial to assess the scale’s convergent validity and internal consistency across the three languages to ensure that the constructs are consistently and accurately measured. Internal consistency was robust across all languages, with Cronbach’s Alpha values ranging from 0.920 to 0.951 and McDonald’s Omega values from 0.940 to 0.960, showing high internal consistency for all factors. CR values for all factors exceeded the acceptable threshold of 0.70, ranging from 0.760 to 0.951, ensuring good convergent validity. Additionally, AVE values, which ranged from 0.547 to 0.736, consistently exceeded the 0.50 threshold, showing an improvement from Experiment 1 and confirming that a substantial portion of variance is explained by the factors across all languages. The results are depicted in Table 5.

**Table 5:** Internal consistency and convergent validity for each factor across the scale’s English, German, and Italian versions tested in Experiment 2.

Factor	No. of items	Internal consistency						Convergent validity					
		English		German		Italian		English		German		Italian	
		$\alpha$	$\omega$	$\alpha$	$\omega$	$\alpha$	$\omega$	CR	AVE	CR	AVE	CR	AVE
Factor 1	10	0.940	0.950	0.920	0.950	0.930	0.940	0.849	0.599	0.760	0.547	0.817	0.554
Factor 2	7	0.945	0.946	0.945	0.946	0.951	0.953	0.945	0.712	0.875	0.590	0.951	0.736
Factor 3	9	0.950	0.960	0.950	0.960	0.950	0.960	0.849	0.669	0.860	0.668	0.878	0.686

Moreover, we examined how the experimental manipulation of a robot’s behavior transparency affected participants’ perceptions. We used two-tailed t-tests and two-way ANOVAs to analyze the data, considering both the experimental manipulation and the participants’ language (English, German, Italian) as factors. We initially planned to include participants’ prior experience with robots as a covariate, but found that language significantly influenced this experience $(F(2, 895) = 5.32, p =0.005, \eta _{\text {p}}^{2} =0.01, 95\% \text { CI } [0.00, 1.00])$, making it unsuitable as a covariate. Results (as shown in Table 6) revealed that participants perceived the robot’s behavior as significantly more transparent in the high transparency condition compared to the low transparency condition $(t(874) = 29.88, p <0.001, 95\% \text { CI } [1.88, 2.14], d = 1.99)$. An ANOVA confirmed this main effect of the transparency manipulation. Additionally, we found a significant main effect of language on perceived transparency. When comparing language groups, we found that German participants perceived the robot as more transparent than English participants $(t(598) = 2.55, p =0.031, 95\% \text { CI } [0.07, 0.53], d = 0.21)$ (Post-hoc t-tests with Bonferroni correction). However, we did not obtain significant differences between English and Italian participants $(t(598) = -2.18, p =0.086, 95\% \text { CI } [-0.48, -0.03], d = -0.18)$, or between German and Italian participants $(t(598) = 0.39, p = 1, 95\% \text { CI } [-0.18, 0.27], d = 0.03)$. There was no significant interaction effect between transparency manipulation and language.

**Fig. 5:**
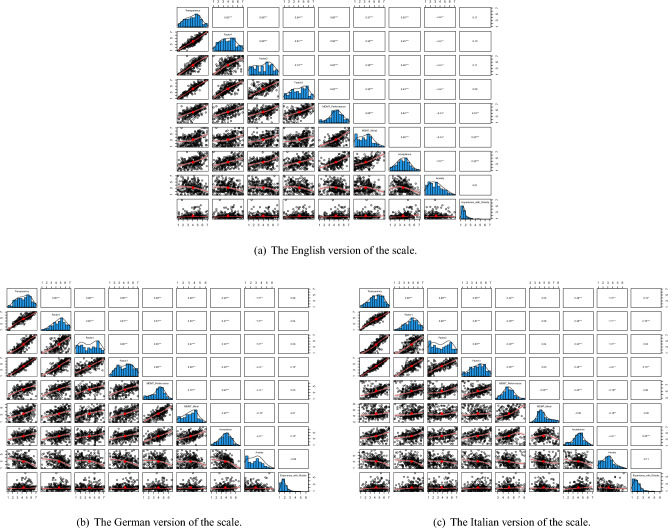
Correlation matrices between the dependent and control variables of Experiment 2 across the three languages versions.

Furthermore, we investigated the effects of the transparency of a robot’s behavior on the three factors of the transparency scale. We also examined whether there were differences between language groups regarding these three factor. For Factor 1, the high transparency condition showed significantly higher scores than the low transparency condition $(t(880) = 16.97, p <0.001, 95\%$ $\text { CI } [1.26, 1.59], d = 1.13)$. An ANOVA (see Table 6) showed that both the manipulated transparency and participants’ language had significant main effects on Factor 1. Post-hoc tests showed that German participants scored lower than English participants $(t(590) = 6.00, p <0.001, 95\% \text { CI } [0.48, 0.94], d = 0.49)$, who scored lower than Italian participants $(t(590) = -5.46, p <0.001, 95\% \text { CI } [-0.87, -0.41], d = -0.45)$. However, there was no significant difference between German and Italian participants $(t(599) = 0.61, p = 1, 95\% \text { CI } [-0.15, 0.28], d = 0.05)$, and no interaction effect was found. For Factor 2, the high transparency condition again scored significantly higher than the low transparency condition $(t(872) = 43.78, p <0.001, 95\% \text { CI } [2.79, 3.06], d = 2.91)$. An ANCOVA confirmed the main effect of manipulated transparency on Factor 2 but found no main effect of language. There was, however, a marginally significant interaction between the transparency manipulation and participant language. Lastly, Factor 3 also showed significantly higher scores in the high transparency condition compared to the low transparency condition $(t(880) = 24.36, p <0.001, 95\% \text { CI } [1.79, 2.11], d = 1.62)$. Finally, the ANOVA confirmed the main effect of the experimental manipulation on Factor 3 but found no significant effect of participant language and no interaction effect. Therefore, the transparency of robot behavior influenced the scale, with some variations based on the language background of the participants. Overall, these results provide support for the sentitivity of the scale to a manipulation of the transparency of a robot’s behavior.

Finally, how the experimental conditions and participants’ language affected other dependent variables was examined (as detailed in Tables S24-S26 of the Supplementary Information). Performance trust was significantly higher in the high transparency condition $(t(857) = 11.06, p <0.001, 95\% \text { CI } [0.75, 1.08], d = 0.73)$. An ANOVA (see Table S24 of the Supplementary Information) showed that both the experimental manipulation and participants’ language had significant effects on performance trust. Post-hoc tests (with Bonferroni correction) revealed that performance trust was significantly lower for the German participants than for the English participants, $t(594) = -3.99$, $p <0.001$, $95\%$ CI $[-0.64, -0.22]$, $d = -0.33$, and for German participants than for Italian participants, $t(584) = -11.77$, $p < 001$, $95\%$ CI $[-1.32, -0.94]$, $d = -0.96$. Furthermore, performance trust was significantly lower for English participants than for Italian participants, $t(569) = -6.98$, $p <.001$, $95\%$ CI $[-0.90, -0.50]$, $d = -0.57$. There was also a significant interaction between experimental manipulation and language. Moral trust was also higher in the high transparency condition $(t(885) = 5.67, p <0.001, 95\% \text { CI } [0.37, 0.75], d = 0.38)$. An ANCOVA (results presented in Table S25 of the Supplementary Information) revealed that the experimental manipulation and participants’ language both had significant effects. Post-hoc tests (with Bonferroni correction) showed that Italian participants had significantly higher moral trust than both English participants,$t(590) = 14.33$, $p <0.001$, $95\%$ CI [1.38, 1.82], $d = 1.17$, and German participants, $t(596) = 15.12$, $p < 001$, $95\%$ CI [1.33, 1.73], $d = 1.23$, while there was no significant difference between English and German participants, $t(576) = 0.67$, $p =0.501$, $95\%$ CI $[-0.14, -0.29]$, $d = 0.05$. An interaction effect was also observed. Robot acceptance was higher in the high transparency condition $(t(884) = 6.53, p <0.001, 95\% \text { CI } [0.36, 0.67], d = 0.44)$. An ANCOVA indicated that both experimental manipulation and language had significant effects (see Table S26 in the Supplementary Information). Post-hoc tests with Bonferroni correction showed that Italian participants had the highest acceptance, significantly higher than both English, $t(590) = 3.96$, $p <0.001$, $95\%$ CI [0.20, 0.60], $d = 0.32$, and German participants, $t(599) = 2.53$, $p =0.048$, $95\%$ CI [0.06, 0.42], $d = 0.21$. There was no significant difference between German and English participants, $t(586) = 1.63$, $p =.289$, $95\%$ CI $[-0.03, 0.36]$, $d = 0.13$. No interaction effect was identified by the ANOVA.

Anxiety towards the robot was lower in the high transparency condition $(t(897) = -4.47, p <0.001, 95\% \text { CI } [-0.56, -0.22], d = -0.30)$. An ANCOVA (as illustrated in Table S27 of the Supplementary Information) showed that both experimental manipulation and language had significant effects. Post-hoc tests (with Bonferroni correction) revealed that German participants had significantly less anxiety than English participants, $t(593) = -2.42$, $p =0.039$, $95\%$ CI $[-0.48, -0.05]$, $d = -0.20$. No significant differences were found between English and Italian participants, $t(588) = 0.49$, $p = 1$, $95\%$ CI $[-0.16, 0.26]$, $d = 0.04$, or between German and Italian participants, $t(597) = -2.08$, $p =0.138$, $95\%$ CI $[-0.42, -0.01]$, $d = -0.17$. There was no interaction effect.

Consistent with Experiment 1, these results confirmed that transparency of a robot’s behavior has a positive influence on trust and acceptance of robots. As seen on Fig. 5, the dimensions of perceived transparency of a robot’s behavior measured by our scale correlated well with trust and acceptance. The consistent pattern of higher transparency corresponding with increased trust and acceptance and decreased anxiety therefore supports the validity of our scale.

**Table 6:** Results of the 2-way ANOVAs conducted on the dependent variables of Experiment 2, with the manipulation of the transparency and the language of participants as independent variables.

Parameter		df	SS	MS	*F* value	*p* value	$\eta p^2$	95% CI
Overall Score of the Perceived Transparency								
Transparency condition		1	910.30	910.30	907.36	< .001 ***	0.50	[0.47, 1.00]
Language		2	19.60	9.80	9.76	< 0.001***	0.02	[0.01, 1.00]
Transparency condition: Language		2	3.60	1.80	1.80	0.167	$<0.01$	[0.00, 1.00]
Residuals		895	897.9	1.0				
Factor 1								
Transparency condition		1	456.40	456.40	306.72	< 0.001 ***	0.26	[0.22, 1.00]
Language		2	98.00	49.00	32.92	< 0.001***	0.07	[0.04, 1.00]
Transparency condition: Language		2	0.80	0.40	0.276	0.759	$<0.01$	[0.00, 1.00]
Residuals		895	1331.90	1.50				
Factor 2								
Transparency condition		1	1925.70	1925.70	1921.13	< 0.001 ***	0.68	[0.66, 1.00]
Language		2	4.60	2.30	2.28	0.103	0.01	[0.00, 1.00]
Transparency condition: Language		2	5.90	3.0	2.94	0.053	0.01	[0.00, 1.00]
Residuals		895	897.10	1.00				
Factor 3								
Transparency condition		1	858.00	858.00	594.44	< 0.001 ***	0.40	[0.36, 1.00]
Language		2	6.30	3.10	2.18	0.114	0.01	[0.00, 1.00]
Transparency condition: Language		2	6.80	3.40	2.36	0.095	0.01	[0.00, 1.00]
Residuals		895	1291.90	1.40				

*df* Degrees of freedom; *SS* Sum of Squares; *MS* Mean Squares.

*p< 0.05; **p< 0.01; ***p< 0.001.

## Discussion

The aim of the present research was to develop and validate a scale to assess the perceived transparency of a robot’s behavior. To do so, we designed a scale reflecting a four-factor model of transparency based on state of the art research on transparency. Accordingly, we distinguish the dimensions legibility, predictability, explainability, and meta-understanding. Contrary to our expectations, Experiment 1 showed that the perceived transparency of a robot consists of three factors. Building upon these findings, we propose the Transparency Of RObots Scale (TOROS), based on a three-factor model: Factor 1, *Illegibility* comprises items expressing difficulty in comprehending the robot’s functioning, objectives, and processes (e.g., “The robot’s overall functioning is a mystery to me”, “I cannot comprehend the robot’s inner processes”). As such, perceived *Illegibility* does not only depend on how legible the actions of a robot are to observers, but also on how they help them infer the robot’s functioning and goals. That is why *Illegibility* encompassed items that were initially assumed to belong to perceived legibility and perceived meta-understanding. *Illegibility* has been chosen instead of legibility because this factor mostly contained reverse-worded items. However, as concluded in Experiment 2, this overrepresentation of reverse-worded items is probably not a research artefact. Indeed, as humans heavily rely on social norms and expectations to understand and socially interact with other agents^69,70^, they are more likely to notice when an agent’s behavior violates or fails to meet such norms or expectations and to engage cognitive resources to understand the agent and the meaning of its behavior^69^. Similarly, when an agent’s communication and actions are unclear, ambiguous, or uninformative, individuals need to spend more cognitive effort to interpret them^70,71^. Therefore, it is possible that the items with the highest factor loading on Factor 1, the highest item discrimination and the lowest item difficulty were mostly reverse-worded because a lack of legibility was a more salient information than legibility for participants to make an evaluation of a robot’s behavior. The Factor 2 *Explainability*, is based on items evaluating perceived quality, clarity, and usefulness of the robot’s explanations about its actions and states (e.g., “The robot explains complex tasks in a way that is easy to understand”, “The robot provides clear explanations for its actions”). The third factor, *Predictability* represents items assessing the users’ ability to anticipate or foresee the robot’s future actions based on its current behavior (e.g., “It is easy for me to foresee the robot’s future actions”, “The robot’s behavior is predictable”). While conceptually related^13–15^, the three proposed TOROS factors offer a new perspective on measuring the perceived robot transparency, supported by empirical data from three countries. In addition, the results showed that these factors were all sensitive to the experimental manipulation of the explainability, legibility, and predictability of a robot’s behavior. Experiment 2 confirmed the factorial structure of the scale in three languages: English, German, and Italian.

Additionally, in both Experiments 1 and 2, the TOROS scale demonstrated a good convergent validity with factors related to transparency, namely trust towards robots and acceptance of robots^16–18^. The experimental manipulation of transparency in both Experiments 1 and 2 had an effect on trust and acceptance and, therefore, confirms that the transparency of a robot’s behavior is an important determinant of trust and acceptance in HRI. Notwithstanding, the effects of the experimental manipulation of transparency on the factors of perceived transparency were the most significant and the largest. Hence, TOROS has the potential to provide more accurate estimations of the influence of perceived transparency on trust, acceptance, and other constructs that transparency is supposed to determine.

Taken together, our results confirm that TOROS represents a reliable and valid measure of the perceived transparency of a robot’s behavior. Besides, it underlies the discrepancy between theorizing about and implementation of transparency in HRI, and how individuals perceive it. More specifically, the results suggest that any manipulation of transparency mostly influences perceived *Explainability*. Interestingly, we found no interaction effect between manipulated explainability, legibility, and predictability of the robot’s behavior on the factors of the scale in Experiment 1, yet all these factors had main effects on the different subscales of the TOROS. This suggests that distinguishing explainability, legibility, and predictability of a robot from perceived transparency of a robot’s behaviors is important. Existing theories of transparency and the factorial structure of TOROS are consistent in terms of what constitutes transparency. Nevertheless, the way transparency is implemented in a robot does not result in equivalent perceptions of transparency in a user’s mind (e.g., In Experiment 1, making a robot more predictable did not only result in higher perceived predictability). This remains to be confirmed in other scenarios. Future research should delve more into the psychological mechanisms that determine the perceived transparency of a robot or any artificial agent.

Interestingly, in Experiment 2, despite TOROS being administered after participants saw an entire video with a robot reaching its goal, an effect of the experimental manipulation of transparency on *Predictability* factor was still detected. This goes against what can be referred to as the *Valley of the normal*: People tend to find ordinary events to be retrospectively predictable^31^. This is due to the fact that understanding processes of resolved events are “backward-looking”: When confronted with unexpected but also unsurprising events, people tend to examine the past in a causal thinking process. This induces them to conclude that such events are self-explanatory and to overestimate their predictability. This phenomenon is known as the hindsight bias^72,73^, and could have skewed the answers of participants. Indeed, as perceived transparency was assessed after a robot’s behavior was fully resolved, a ceiling effect for *Predictability* could have been observed, and yet was not. Further examination of this phenomenon in future research is required.

TOROS is the first reliable and valid tool to measure perceived transparency of robots’ behavior. The scale and its instructions in all three languages are provided in the supplementary material. Researchers can use it to test the influence of selected independent variables on perceived transparency. These independent variables can be features of a robot’s behavior (e.g., social cues, structure or content of a robot’s utterances), contextual factors that may affect users’ ability to interpret the robot behavior (e.g., task difficulty, noisiness of the surrounding environment), individual factors that could affect how people interpret and evaluate the transparency of a robot (e.g., existing attitudes towards robots), or a potential interaction between such variables. TOROS can also be used to assess how perceived transparency determines or correlates with other factors (such as trust or acceptance). Because of the multidimensionality of the scale, researchers can select the dimensions of perceived transparency they specifically intend to explore by using factors of interest as subscales. In addition, the high sensitivity of TOROS to manipulations of transparency, as shown in Experiments 1 and 2, makes TOROS very relevant as a manipulation check to verify whether an experimental manipulation of transparency was successful. Ultimately, the purpose of TOROS is to provide researchers with a validated tool to limit reliance on self-made and non-tested items, as seen in prior research^23^. As such, TOROS will improve the internal validity and reliability of future empirical results, while improving their comparability by establishing a measurement standard in research on transparency. For TOROS to efficiently and accurately measure perceived transparency of robots’ behavior, it must be used as intended. This includes using the same fully labelled 7-point Likert format^42^ and the exact same items, as well as randomizing the order of all items^50,74^, or at least alternate between positively worded and reverse-worded items to hinder both acquiescence bias and careless responding^40^. If other constructs are measured alongside perceived transparency and the order of the measures does not matter, the items can be mixed together and randomized as well^40,50,74^. Any change of the scale’s format, rephrasing of item, or removal of the items should be avoided, or done with strong theoretical or methodological rationales. In case of changes due to justified research needs, the validity and reliability of the scale should be assessed again, at least by conducting a CFA and calculation of internal consistency, to make sure such adjustments did not negatively impact the validity and reliability of the scale. Some researchers may be interested in calculating a composite score for perceived transparency of robots’ behavior. As suggested by the CFAs of Experiment 2, perceived transparency of a robot’s behavior is not a one-factor construct, and should preferably be addressed as a three-dimensional construct. However, the results of both Experiments 1 and 2 provided support for the sensitivity of the composite score to manipulations of the transparency of a robot’s behavior. To calculate a composite score of perceived transparency, the intended coding of the items measuring perceived *Illegibility* should be reversed (i.e., the two items supposed to be reverse-coded should be left unchanged, while the eight items supposed to be left unchanged should be reverse-coded), so its polarity is consistent with perceived *Explainability* and perceived *Predictability*. Once this is achieved, an average of the three subscores of TOROS can be calculated, but should be interpreted with caution, while acknowledging and justifying this scoring as a methodological choice. A CFA based on a 1-Factor model can be considered to assess the validity of this approach in the context of a new study, but is likely to result in fit indices that are as poor as those obtained in Experiment 2.

Despite the promising results of the TOROS scale, the present research does not come without methodological limitations: For instance, a crucial limitation pertains to the measurement invariance results across the different language versions of the scale. Even though configural and metric invariance were achieved, TOROS did not demonstrate full scalar and residual invariance. Besides, there were slight differences in perceived transparency across languages, particularly regarding English and German participants, as well as between English and Italian participants. However, it is important to note that achieving only partial measurement invariance is not uncommon in cross-cultural research, especially when dealing with complex psychological constructs like transparency in HRI. Indeed, partial invariance can still allow for meaningful cross-group comparisons^75^. The strong internal consistency and convergent validity demonstrated across all three language versions suggest that the scale is reliable and valid within each language context. Additionally, the observed differences between languages in terms of perceived transparency were small, and almost no interaction between the language of the participant and the manipulation of transparency was observed. However, further research should investigate cultural factors that may affect how people construe transparency in robots. As such inquiries would require TOROS to be available in the language of the targeted cultures, validations of new translations should be conducted, either before or during these studies. Our current methodology aligns well with established practices in scale development in HRI^25,28,32,76^. Thus, image vignettes and videos of real scenarios provided a strong foundation for scale development. Going beyond such classic approach, we recommend to conduct further validation research in the HRI context, in particular on participants directly interacting with robots (in constrast with participants observing them). We look forward to seeing the TOROS scale put to use to shed further light on the notion of transparency in social robots.

## Conclusions

As robots become more sophisticated and prevalent in our society, the need to measure how transparent they are to humans becomes increasingly important. Yet, until now, a standardized method to measure this critical aspect of robot behavior has been lacking. This work addresses this gap by developing and validating the first comprehensive scale to assess the perceived transparency of robotic systems, termed TOROS. Through a rigorous three-stage process involving 1,223 participants, we have created a robust tool encompassing 26 items and comprised of three factors: *Illegibility*, *Explainability*, and *Predictability*. The scale demonstrates high cross-linguistic reliability and validity across English, German, and Italian languages. We believe that the proposed scale can serve as a valuable tool in various HRI experiments to examine the effects of transparency-related aspects on other phenomena. For instance, it can be employed in studies of human-robot collaboration to evaluate how transparency impacts team performance, in learning and adaptation research to track changes in the understanding of the robot as users gain experience with it, and in error recovery and management to assess the effectiveness of error handling strategies. The scale can also be used to investigate how different robot designs and behaviors influence perceived transparency and how this, in turn, affects the overall interaction quality.

Future research should focus on translating the scale into more languages and examining its performance in real HRI scenarios or in interaction with different artificial agents. Follow-up works can use the scale to understand the determinants of perceived transparency in HRI and contribute to a better understanding of the psychological mechanisms at play. This tool opens up new avenues for research and has the potential to significantly enhance our understanding of transparency in robotics, ultimately leading to the development of more effective and user-friendly robotic systems.

## Supplementary Information

Supplementary Information 1.

Supplementary Information 2.

Supplementary Information 3.

Supplementary Information 4.

## Data Availability

The datasets generated and analyzed during the current study are available from the corresponding authors upon request.
