# Supplementary material for: Measuring transparency in intelligent robots
Source: Sci Rep. 2025 Dec 12;15:43809. doi: 10.1038/s41598-025-29685-w (PMC12705673; doi:10.1038/s41598-025-29685-w)
Supplement: Supplementary file 1 — Supplementary Information 1. [file 41598_2025_29685_MOESM1_ESM.pdf]

Measuring Transparency in Intelligent Robots

Georgios Angelopoulos[1,*,+], Dimitri Lacroix[2,,+], Ricarda Wullenkord[2], Alessandra Rossi[1], Silvia Rossi[1], and Friederike Eyssel[2]**

[1]Interdepartmental Center for Advances in Robotic Surgery - ICAROS, University of Naples Federico II, Naples, 80131, Italy
[2]Center for Cognitive Interaction Technology - CITEC, Bielefeld University, Bielefeld, 33619, Germany
[*]georgios.angelopoulos@unina.it
[**]dimitri.lacroix@uni-bielefeld.de
[+]these authors contributed equally to this work

Supplementary Information

Item Generation

ID	Subscale	Item
TR1	**Legibility**	It is clear to me what the robot does.
TR2		I can easily understand the robot's actions.
TR3		I can quickly grasp the task the robot performs.
TR4		I find the robot's behavior easy to understand.
TR5		I feel informed about the robot's activities.
TR6		I do not understand the robot's actions.
TR7		The robot's behavior is confusing to me
TR8		I am unsure what the robot does.
TR9		The robot's actions are obvious.
TR10		The robot provides information about its actions.
TR11		The robot's cues provide relevant information about what it does.
TR12		The robot's behavior makes sense.
TR13		The robot's behavior is legible
TR14		The robot's actions are hard to follow.
TR15		The robot's behavior is difficult to read.
TR16		It is impossible to know what the robot does.
TR17	**Predictability**	I can predict what the robot will do next.
TR18		The robot's next steps are clear to me.
TR19		I feel confident in predicting the robot's next moves.
TR20		It is easy for me to foresee the robot's future actions.
TR21		I see the pattern in the robot's behavior.
TR22		I cannot anticipate what the robot's goal is.
TR23		It is difficult for me to tell what the robot will do next.
TR24		I feel that the robot is inconsistent.
TR25		The robot's actions are consistent.
TR26		The robot's behavior is predictable.
TR27		The robot provides cues that help predict its next actions.
TR28		It is easy to anticipate what will follow the robot's behavior.
TR29		The robot's past actions help to predict its future behavior.
TR30		The robot's behavior does not help predict what it will do next.
TR31		The robot's actions are unpredictable.
TR32		The robot acts in a way that is random.
TR33	**Explainability**	I find the robot's explanations informative.
TR34		I understand why the robot performs its actions.
TR35		The robot's explanations make its actions clear to me.
TR36		I feel like the robot's explanations are useful.
TR37		I feel like the robot's explanations are necessary to understand its actions.
TR38		I find the robot's explanations incoherent.
TR39		The robot's explanations do not make sense to me.
TR40		I cannot explain the robot's behavior.
TR41		The robot provides clear explanations for its actions.
TR42		The robot's behavior is explainable
TR43		The robot provides detailed explanations of its actions.
TR44		The robot's explanations for its actions are straightforward.
TR45		The robot explains complex tasks in a way that is easy to understand.
TR46		The robot's explanations are overly detailed.
TR47		The robot provides unnecessary explanations.
TR48		The robot does not provide enough explanations.
TR49	**Meta Understanding**	The robot's behavior helps me understand its objectives.
TR50		I have a clear understanding of how the robot operates in general.
TR51		I feel like the robot helps me understand its inner processes.
TR52		I am confident in understanding of the robot's overall behavior.
TR53		The robot provides enough cues for me to understand its overall functioning.
TR54		I cannot comprehend the robot's inner processes.
TR55		The robot's overall functioning is a mystery to me.
TR56		I am confused about the robot's general objectives.
TR57		The robot conveys its overall state effectively.
TR58		The robot ensures that its users are well-informed about its activities.
TR59		The robot's functioning is transparent.
TR60		The clarity of the robot's overall functioning eliminates any doubts about it.
TR61		The robot's inner processes are obvious.
TR62		The robot does not provide enough information about its overall objectives.
TR63		It is hard to make sense of the robot's general functioning.
TR64		It is difficult to get a clear picture of the robot's overall operations.

Table S1. The 64 initial items of the scale.

For ease of reference, each item in the scale was assigned a unique identifier, ranging from TR1 to TR64, where 'TR' stands for 'Transparency' and the number indicates the item's position in the scale.

Pre-test

Before Experiment 1, we conducted a *pretest* to identify a hypothetical everyday life scenario featuring HRI that would effectively discriminate transparency. This way, we maximize the scale's sensitivity and effectiveness for measuring transparency. Consequently, we developed four image vignettes, each depicting a robot performing a task in an everyday life scenario (i.e., Scenario 1: A robot heading towards a charging station to refill its battery; Scenario 2: A robot taking an apple core to throw it in a bin; Scenario 3: Arobot switching the light on; and Scenario 4: A robot taking a guest's coat to put in on a rack). The pretest was implemented on Qualtrics and employed a $4 \times 2 \times 2 \times 2$ mixed between-within-subjects design that manipulated explainability, legibility, and predictability of a robot's behavior as a between-subjects factor and treated the context of the scenario as a within-subject factor, resulting in 32 images vignettes total. Transparency was assessed with three items that evaluated the explainability, legibility, and predictability of the respective robot's behavior. Additionally, one item evaluated the understandability of the picture vignettes, and an attention check was implemented at the study's beginning. Furthermore, demographic questions (i.e., age, gender, education, self-assessed English language proficiency) and prior experience with robots. Only complete datasets from participants over 18 years of age and with a self-declared English proficiency at A2 level (Elementary) and above were included. The pretest was designed to run for two weeks or to be discontinued if a sample size of 160 participants was reached before the end of this period. The two-week duration was chosen to balance the need for timely data collection and to allow for sufficient time to recruit participants. Participants for the pretest were recruited via mailing lists, social media, and snowball sampling. After analyzing the pretest, one scenario with eight image vignettes was selected for further use, based on the greatest contrast of mean between high and low transparency. The pretest's pre-registration details are available at https://aspredicted.org/ZTT_STV.

As outlined in our pre-registered criteria, the pretest stopped after two weeks, allowing us to recruit 97 participants through social media and snowball sampling. Following our pre-registered exclusion criteria, 31 participants were disqualified; 24 failed to complete the study, 6 were excluded for not passing both attention checks, and 1 was removed upon request for data removal. Thus, the final sample comprised $N = 66$ participants.

Pretest results revealed that Scenario 1 (i.e., a robot heading towards a charging station to refill its battery) elicited the most substantial variation in responses across all dependent variables, particularly in the measures of explainability and predictability, as depicted in Table S2. This variation, as evident in descriptive statistics such as means and standard deviations, indicates that Scenario 1 elicited a greater range in participant responses, making it the most effective in distinguishing among the different conditions tested. For example, for Scenario 1, the mean scores for transparency were lower when explainability, legibility, and predictability were absent ($M = 3.500$, $SD = 1.225$), which is consistent with expectations. Conversely, higher mean scores for transparency were observed in Scenario 1 when all three factors were present ($M = 6.200$, $SD = 1.135$), demonstrating the scenario's sensitivity to the manipulations. Additionally, we observed that three participants in the low transparency condition (low explainability, low predictability, and low explainability, respectively) reported being able to anticipate the robot's objectives, as these were accentuated through color highlighting, thereby enhancing the robot's predictability and the salience of its goal. Based on these findings, we selected Scenario 1. The sole modification implemented in Scenario 1 involved extending the color application to the entire environment rather than restricting it to specific objects. This modification was hypothesized to amplify the distinction between the conditions within Scenario 1 without altering the relative effectiveness of the manipulations across different scenarios. Therefore, at this point, it was decided to proceed with Experiment 1.

ID	Scenario			Dependent Variables							
	Manipulation			Explainability		Predictability		Legibility		Transparency	
	Explainability	Predictability	Legibility	Mean	Std dev	Mean	Std dev	Mean	Std dev	Mean	Std dev
1	NO	NO	NO	4.17	1.329	3.83	1.472	4.17	1.329	3.50	1.225
	NO	NO	YES	5.90	1.853	5.00	1.700	5.50	1.900	5.10	1.729
	NO	YES	NO	5.38	2.200	5.25	2.435	5.63	2.326	5.50	2.262
	YES	NO	NO	4.33	2.338	3.67	1.966	4.17	2.483	4.00	2.280
	NO	YES	YES	6.00	2.236	5.29	2.430	5.86	2.193	5.14	2.116
	YES	YES	NO	7.00	.000	7.00	.000	6.67	.707	6.78	.667
	YES	NO	YES	6.00	1.155	6.10	1.101	5.80	1.317	5.60	1.506
	YES	YES	YES	6.60	.966	6.00	1.414	6.30	.949	6.20	1.135
2	NO	NO	NO	4.83	2.137	4.83	2.137	5.17	1.835	5.00	2.000
	NO	NO	YES	6.30	.949	5.90	1.449	5.50	1.354	6.00	1.155
	NO	YES	NO	5.63	2.326	5.63	2.200	5.75	2.053	5.25	2.375
	YES	NO	NO	4.33	2.160	4.67	1.211	4.83	2.401	4.83	2.280
	NO	YES	YES	6.14	.900	5.71	1.496	6.14	1.069	5.71	1.380
	YES	YES	NO	6.33	1.000	6.44	.882	6.33	.866	6.11	.928
	YES	NO	YES	5.30	1.418	5.00	.943	5.40	1.265	4.80	1.506
	YES	YES	YES	6.00	1.247	5.70	1.059	5.80	1.398	5.50	1.269
3	NO	NO	NO	4.83	1.941	4.83	1.941	4.67	1.751	5.00	2.098
	NO	NO	YES	4.10	2.378	4.10	2.132	4.40	2.221	3.70	2.058
	NO	YES	NO	5.37	2.264	5.38	2.722	5.88	2.031	5.50	2.070
	YES	NO	NO	4.83	1.722	4.67	1.506	5.17	1.722	5.17	1.941
	NO	YES	YES	5.43	1.813	4.57	2.299	5.71	1.799	4.43	2.370
	YES	YES	NO	5.11	1.691	5.44	2.128	5.22	2.048	5.00	1.803
	YES	NO	YES	5.80	1.398	5.30	1.767	5.50	1.780	5.70	1.494
	YES	YES	YES	5.60	1.506	5.30	1.636	5.60	1.075	5.50	1.179
4	NO	NO	NO	5.17	1.835	5.17	2.137	5.17	.983	5.00	2.000
	NO	NO	YES	6.00	1.155	5.70	1.252	5.20	1.398	5.70	1.160
	NO	YES	NO	5.00	2.070	4.13	2.167	4.00	2.268	4.13	1.885
	YES	NO	NO	4.33	1.966	3.83	1.835	5.33	1.633	5.00	1.673
	NO	YES	YES	6.57	.535	6.43	.787	5.71	1.380	5.86	1.345
	YES	YES	NO	6.11	1.691	4.11	1.965	5.44	1.878	5.56	1.236
	YES	NO	YES	4.60	1.713	4.60	1.350	4.70	1.703	4.70	1.494
	YES	YES	YES	4.80	1.814	4.50	1.958	4.60	1.838	4.80	1.476

Table S2. The results of pre-test.

Experiment 1

Conditions (C1-C8)	No. of Participants	Age		Gender			Experience with robots	
		M	SD	Male	Female	Other	M	SD
C1 - **High Explainability**, **High Legibility**, **High Predictability**	40	27.88	7.95	17	22	1	2.04	1.05
C2 - **High Explainability**, **High Legibility**, Low Predictability	38	31.37	10.24	15	22	1	2.17	1.19
C3 - **High Explainability**, Low Legibility, **High Predictability**	43	28.63	7.86	15	28	0	1.97	1.10
C4 - Low Explainability, **High Legibility**, **High Predictability**	39	30.69	7.88	21	18	0	1.88	1.00
C5 - **High Explainability**, Low Legibility, Low Predictability	38	32.61	11.50	15	23	0	2.14	1.23
C6 - Low Explainability, **High Legibility**, Low Predictability	41	28.73	11.35	19	20	2	2.30	1.27
C7 - Low Explainability, Low Legibility, **High Predictability**	43	30.53	9.72	16	27	0	2.05	1.17
C8 - Low Explainability, Low Legibility, Low Predictability	40	28.4	7.03	17	22	1	2.08	1.03
**Total**	**322**	**29.82**	**9.34**	**135**	**182**	**5**	**2.08**	**1.13**

Table S3. Demographic information across the eight conditions.

Initial item removals, including TR37, TR46, and TR47, were excluded due to low loadings in the initial factor structure matrix. Following this removal, a new EFA indicated the need to exclude TR4, TR6, TR7, TR11, TR15, TR21, TR22, TR29, TR34, TR39, TR49, TR52, TR59, TR60 due to insufficient loadings, and TR62 for loading on multiple factors. Further iterations excluded TR3, TR25, TR42, and TR61 due to low loadings, and TR2 and TR12 for constituting underrepresented factors. The fourth iteration led to removing TR14 and TR24 due to inadequate loadings, TR31 for multiple factor loadings, and TR32 and TR38 for being in underrepresented factors. The fifth iteration led to the removal of TR48 for loading on various factors and TR51 and TR53 due to low loadings. Ultimately, a final iteration confirmed that no further items required removal.

Items	Initial	Extraction	Items	Initial	Extraction
It is clear to me what the robot does.	.790	.744	I can quickly grasp the task the robot performs.	.734	.637
I feel informed about the robot's activities.	.765	.705	I find the robot's behavior easy to understand.	.789	.734
I am unsure what the robot does.	.719	.617	I do not understand the robot's actions.	.752	.673
The robot's actions are obvious.	.719	.604	The robot's behavior is confusing to me.	.730	.661
The robot provides information about its actions.	.781	.738	The robot's cues provide relevant information about what it does.	.679	.560
It is impossible to know what the robot does.	.687	.594	The robot's behavior makes sense.	.755	.703
I can predict what the robot will do next.	.747	.700	The robot's behavior is legible.	.692	.601
The robot's next steps are clear to me.	.760	.707	The robot's actions are hard to follow.	.693	.598
I feel confident in predicting the robot's next moves.	.741	.685	The robot's behavior is difficult to read.	.746	.648
It is easy for me to foresee the robot's future actions.	.699	.630	I see the pattern in the robot's behavior.	.646	.523
It is difficult for me to tell what the robot will do next.	.715	.681	I cannot anticipate what the robot's goal is.	.654	.561
The robot's behavior is predictable.	.760	.699	I feel that the robot is inconsistent.	.553	.402
The robot provides cues that help predict its next actions.	.672	.549	The robot's actions are consistent.	.662	.547
It is easy to anticipate what will follow the robot's behavior.	.686	.633	The robot's past actions help to predict its future behavior.	.467	.325
The robot's behavior does not help predict what it will do next.	.617	.523	The robot's actions are unpredictable.	.748	.674
I find the robot's explanations informative.	.796	.761	The robot acts in a way that is random.	.609	.471
The robot's explanations make its actions clear to me.	.817	.764	I understand why the robot performs its actions.	.775	.680
I feel like the robot's explanations are useful.	.746	.698	I feel like the robot's explanations are necessary to understand its actions.	.405	.247
I cannot explain the robot's behavior.	.739	.642	I find the robot's explanations incoherent.	.575	.520
The robot provides clear explanations for its actions.	.753	.695	The robot's explanations do not make sense to me.	.588	.518
The robot provides detailed explanations of its actions.	.705	.636	The robot's behavior is explainable.	.688	.618
The robot's explanations for its actions are straightforward.	.800	.736	The robot's explanations are overly detailed.	.428	.240
The robot explains complex tasks in a way that is easy to understand.	.735	.690	The robot provides unnecessary explanations.	.332	.195
I have a clear understanding of how the robot operates in general.	.688	.637	The robot does not provide enough explanations.	.700	.658
I cannot comprehend the robot's inner processes.	.603	.544	The robot's behavior helps me understand its objectives.	.788	.734
The robot's overall functioning is a mystery to me.	.650	.582	I feel like the robot helps me understand its inner processes.	.677	.597
I am confused about the robot's general objectives.	.711	.601	I am confident in understanding of the robot's overall behavior.	.769	.698
The robot conveys its overall state effectively.	.647	.569	The robot provides enough cues for me to understand its overall functioning.	.753	.689
The robot ensures that its users are well-informed about its activities.	.760	.711	The robot's functioning is transparent.	.723	.640
It is hard to make sense of the robot's general functioning.	.635	.567	The clarity of the robot's overall functioning eliminates any doubts about it.	.701	.635
It is difficult to get a clear picture of the robot's overall operations.	.636	.563	The robot's inner processes are obvious.	.675	.504
I can easily understand the robot's actions.	.790	.741	The robot does not provide enough information about its overall objectives.	.686	.623

Table S4. The communalities of the 64 initial items.

Item	Factor				
	1	2	3	4	5
I find the robot's behavior easy to understand.	**.837**	-.013	.095	.058	.144
The robot's behavior helps me understand its objectives.	**.834**	.106	.094	.035	.129
I can easily understand the robot's actions.	**.829**	-.013	.112	.097	.177
I am confident in understanding of the robot's overall behavior.	**.816**	.059	.033	-.161	.029
The robot's explanations make its actions clear to me.	**.813**	.267	-.038	.138	-.106
It is clear to me what the robot does.	**.811**	.042	-.095	-.174	.213
The robot provides enough cues for me to understand its overall functioning.	**.808**	.164	-.018	-.096	-.024
I feel informed about the robot's activities.	**.799**	.227	-.109	.046	.012
The robot's explanations for its actions are straightforward.	**.798**	.221	-.016	.161	-.155
The robot's next steps are clear to me.	**.793**	-.005	.278	-.019	.016
I understand why the robot performs its actions.	**.789**	-.046	.038	.076	.219
The robot's functioning is transparent.	**.781**	.085	-.062	-.103	.095
The robot ensures that its users are well-informed about its activities.	**.772**	.312	-.114	.040	-.059
The robot's behavior makes sense.	**.771**	-.079	.072	.197	.243
The robot provides information about its actions.	**.766**	.351	-.043	.106	-.125
I find the robot's explanations informative.	**.765**	.358	-.096	.158	-.115
The robot provides clear explanations for its actions.	**.763**	.268	-.055	.095	-.168
I can quickly grasp the task the robot performs.	**.763**	.022	.067	-.093	.204
The robot's behavior is difficult to read.	**-.762**	.242	.074	-.034	-.041
The clarity of the robot's overall functioning eliminates any doubts about it.	**.761**	.166	-.038	-.157	.043
I do not understand the robot's actions.	**-.755**	.248	.116	-.134	-.096
The robot's behavior is legible.	**.750**	.030	.098	.134	.100
The robot does not provide enough explanations.	**-.747**	-.014	.280	.016	.145
The robot's behavior is predictable.	**.744**	-.086	.362	-.053	-.068
I feel confident in predicting the robot's next moves.	**.741**	-.092	.342	-.081	-.067
The robot's actions are obvious.	**.737**	.003	.239	.053	.017
The robot's behavior is explainable.	**.737**	-.031	.060	.143	.222
I have a clear understanding of how the robot operates in general.	**.727**	.132	-.071	-.284	.070
The robot's cues provide relevant information about what it does.	**.726**	.160	-.007	-.003	.086
The robot's behavior is confusing to me.	**-.726**	.284	.110	-.088	-.185
I feel like the robot helps me understand its inner processes.	**.721**	.235	-.072	-.115	-.063
The robot does not provide enough information about its overall objectives.	**-.721**	.032	.263	.107	.151
I am unsure what the robot does.	**-.719**	.170	.223	.112	-.090
I cannot explain the robot's behavior.	**-.717**	.347	.069	.040	-.031
The robot explains complex tasks in a way that is easy to understand.	**.717**	.401	-.116	-.039	-.003
It is impossible to know what the robot does.	**-.715**	.228	.174	.001	-.024
I can predict what the robot will do next.	**.712**	-.093	.411	-.069	-.107
I am confused about the robot's general objectives.	**-.709**	.092	.244	.172	-.021
I see the pattern in the robot's behavior.	**.705**	.038	.137	.035	.070
I cannot anticipate what the robot's goal is.	**-.704**	.218	-.022	.122	.056
It is easy to anticipate what will follow the robot's behavior.	**.704**	-.063	.293	-.136	-.171
The robot conveys its overall state effectively.	**.702**	.189	-.072	.185	-.031
The robot provides cues that help predict its next actions.	**.702**	.095	.203	.029	-.073
It is difficult for me to tell what the robot will do next.	**-.700**	.296	-.158	.111	.256
The robot's actions are consistent.	**.699**	-.016	.203	.107	.077
I feel like the robot's explanations are useful.	**.696**	.376	-.136	.197	-.118
The robot's actions are hard to follow.	**-.690**	.283	-.030	-.200	.006
The robot's actions are unpredictable.	**-.688**	.366	-.119	-.025	.228
The robot provides detailed explanations of its actions.	**.677**	.402	-.036	-.075	-.097
The robot's overall functioning is a mystery to me.	**-.673**	.209	.237	.164	-.044
It is easy for me to foresee the robot's future actions.	**.673**	-.084	.388	-.112	-.085
The robot's explanations do not make sense to me.	**-.659**	.116	.161	-.207	.022
It is difficult to get a clear picture of the robot's overall operations.	**-.658**	.124	.219	.256	.018
It is hard to make sense of the robot's general functioning.	**-.655**	.211	.274	.131	.021
The robot's inner processes are obvious.	**.652**	.103	.030	-.258	-.011
The robot's behavior does not help predict what it will do next.	**-.652**	.229	-.130	.001	.166
I cannot comprehend the robot's inner processes.	**-.629**	.220	.222	.199	.107
The robot acts in a way that is random.	**-.536**	.408	.063	-.112	.026
The robot's past actions help to predict its future behavior.	**.530**	.071	.129	.065	.135
I feel that the robot is inconsistent.	**-.521**	.326	.115	-.093	.056
I find the robot's explanations incoherent.	**-.509**	.318	.186	-.336	.111
The robot's explanations are overly detailed.	.150	.407	-.035	-.206	.089
I feel like the robot's explanations are necessary to understand its actions.	.283	.296	-.025	.280	-.013
The robot provides unnecessary explanations.	-.197	.262	.185	-.199	.118

Table S5. The factor structure matrix with the 64 initial items of the scale.

Item	Factor				
	1	2	3	4	5
The robot's behavior makes sense.	**.651**	-.122	-.004	.115	-.106
The robot's behavior is explainable.	**.585**	-.064	-.041	.131	-.103
I understand why the robot performs its actions.	**.573**	-.038	-.137	.103	-.124
The robot's behavior is confusing to me.	**-.535**	.246	.287	.043	.019
I can easily understand the robot's actions.	**.534**	-.029	-.056	.157	-.236
I can quickly grasp the task the robot performs.	**.480**	.127	-.236	.049	-.197
I find the robot's behavior easy to understand.	**.475**	-.020	-.106	.161	-.257
It is clear to me what the robot does.	**.460**	.146	-.456	.083	-.048
I do not understand the robot's actions.	**-.426**	.296	.250	-.083	.064
The robot's behavior helps me understand its objectives.	**.424**	.063	-.100	.261	-.244
The robot's behavior is legible.	**.405**	-.058	-.003	.246	-.229
The robot's actions are consistent.	.368	-.039	.070	.149	-.367
The robot's past actions help to predict its future behavior.	.367	.054	.033	.137	-.177
I see the pattern in the robot's behavior.	.321	.020	-.040	.181	-.310
The robot's behavior is difficult to read.	-.319	.230	.300	-.049	.186
I find the robot's explanations incoherent.	-.130	**.566**	.075	-.225	.013
The robot provides unnecessary explanations.	.022	**.417**	.095	-.095	-.053
The robot's explanations are overly detailed.	.031	**.405**	-.165	.233	.090
The robot acts in a way that is random.	-.214	.393	.194	.081	.190
I feel that the robot is inconsistent.	-.142	.357	.233	-.011	.135
The robot's actions are hard to follow.	-.305	.353	.052	-.085	.269
The robot's explanations do not make sense to me.	-.227	.320	.165	-.304	.015
It is difficult to get a clear picture of the robot's overall operations.	-.075	.016	**.623**	-.029	.101
I cannot comprehend the robot's inner processes.	.026	.148	**.585**	-.029	.159
The robot's overall functioning is a mystery to me.	-.209	.115	**.583**	.015	.030
I am confused about the robot's general objectives.	-.164	.052	**.579**	-.110	.025
It is hard to make sense of the robot's general functioning.	-.118	.175	**.579**	-.052	.022
I am unsure what the robot does.	-.301	.109	**.530**	-.031	.000
The robot does not provide enough information about its overall objectives.	.068	.135	**.523**	-.321	.100
I have a clear understanding of how the robot operates in general.	.183	.235	**-.485**	.149	-.168
The robot does not provide enough explanations.	.031	.177	**.455**	-.428	.044
It is impossible to know what the robot does.	-.257	.232	**.400**	-.071	.080
I cannot explain the robot's behavior.	-.286	.241	.371	.099	.235
The robot's inner processes are obvious.	.070	.204	-.357	.131	-.316
The clarity of the robot's overall functioning eliminates any doubts about it.	.194	.164	-.346	.273	-.186
The robot's functioning is transparent.	.302	.088	-.345	.218	-.129
I am confident in understanding of the robot's overall behavior.	.215	.115	-.327	.177	-.321
I feel like the robot's explanations are useful.	.017	-.047	-.052	**.791**	-.008
I find the robot's explanations informative.	.035	-.022	-.074	**.757**	-.091
The robot provides information about its actions.	.012	.024	-.076	**.705**	-.176
The robot provides clear explanations for its actions.	-.041	-.039	-.112	**.657**	-.217
The robot's explanations make its actions clear to me.	.077	-.044	-.075	**.653**	-.192
The robot's explanations for its actions are straightforward.	.020	-.099	-.041	**.645**	-.254
The robot ensures that its users are well-informed about its activities.	.084	.050	-.207	**.617**	-.080
The robot explains complex tasks in a way that is easy to understand.	.107	.184	-.245	**.595**	-.022
The robot provides detailed explanations of its actions.	-.041	.201	-.193	**.591**	-.180
The robot conveys its overall state effectively.	.177	-.098	-.052	**.544**	-.067
I feel like the robot's explanations are necessary to understand its actions.	.102	-.050	.211	**.538**	.091
I feel informed about the robot's activities.	.211	.021	-.225	**.509**	-.061
I feel like the robot helps me understand its inner processes.	.030	.126	-.309	**.426**	-.183
The robot provides enough cues for me to understand its overall functioning.	.134	.096	-.287	.358	-.254
The robot's cues provide relevant information about what it does.	.301	.081	-.177	.313	-.128
I can predict what the robot will do next.	.087	.030	.080	.018	**-.816**
It is easy for me to foresee the robot's future actions.	.089	.070	.035	-.019	**-.774**
It is easy to anticipate what will follow the robot's behavior.	-.047	.034	-.070	.083	**-.746**
The robot's behavior is predictable.	.151	.021	.043	.037	**-.736**
I feel confident in predicting the robot's next moves.	.141	.032	.001	.020	**-.723**
It is difficult for me to tell what the robot will do next.	.135	.201	.212	.004	**.707**
The robot's actions are unpredictable.	.047	.339	.140	-.010	**.613**
The robot's next steps are clear to me.	.273	.049	.002	.121	**-.565**
The robot's behavior does not help predict what it will do next.	-.001	.208	.115	-.060	**.541**
The robot provides cues that help predict its next actions.	.111	.024	.030	.293	**-.478**
The robot's actions are obvious.	.277	-.005	.048	.169	**-.476**
I cannot anticipate what the robot's goal is.	-.120	.110	.329	.018	.391

Table S6. The factor pattern matrix with the 64 initial items of the scale.

Item	Factor				
	1	2	3	4	5
The robot's explanations make its actions clear to me.	**.831**	.286	.049	-.143	-.050
The robot's explanations for its actions are straightforward.	**.817**	.244	.067	-.169	-.066
I feel informed about the robot's activities.	**.805**	.205	-.084	.058	.054
The robot's next steps are clear to me.	**.797**	-.125	.246	.050	.100
It is clear to me what the robot does.	**.796**	-.026	-.162	.165	.154
The robot provides information about its actions.	**.789**	.331	.032	-.045	-.008
I find the robot's explanations informative.	**.789**	.362	-.004	-.076	-.002
The robot provides clear explanations for its actions.	**.785**	.273	.031	-.054	.014
The robot ensures that its users are well-informed about its activities.	**.781**	.311	-.054	.046	-.018
I feel confident in predicting the robot's next moves.	**.744**	-.227	.279	.051	-.084
The robot explains complex tasks in a way that is easy to understand.	**.739**	.323	-.072	.150	.113
I have a clear understanding of how the robot operates in general.	**.738**	-.005	-.163	.264	-.148
The robot's behavior is predictable.	**.733**	-.181	.310	.046	-.044
The robot's actions are obvious.	**.724**	-.043	.214	-.031	.032
I can predict what the robot will do next.	**.723**	-.252	.360	.030	-.006
I feel like the robot's explanations are useful.	**.720**	.422	-.042	-.125	-.060
It is easy to anticipate what will follow the robot's behavior.	**.717**	-.202	.256	.018	-.084
It is difficult for me to tell what the robot will do next.	**-.709**	.363	-.130	.134	.046
The robot provides cues that help predict its next actions.	**.708**	.016	.247	.081	.218
I am unsure what the robot does.	**-.706**	.197	.282	.022	-.231
The robot conveys its overall state effectively.	**.705**	.222	-.007	-.090	.058
I cannot explain the robot's behavior.	**-.705**	.359	.161	.162	-.067
The robot provides detailed explanations of its actions.	**.703**	.307	.025	.159	-.112
I am confused about the robot's general objectives.	**-.702**	.134	.317	.000	.204
It is impossible to know what the robot does.	**-.697**	.201	.224	.097	-.021
It is easy for me to foresee the robot's future actions.	**.678**	-.228	.331	.073	-.044
It is difficult to get a clear picture of the robot's overall operations.	**-.669**	.184	.305	-.117	.048
The robot's overall functioning is a mystery to me.	**-.664**	.229	.333	-.020	.085
The robot's behavior does not help predict what it will do next.	**-.643**	.259	-.092	.115	.008
It is hard to make sense of the robot's general functioning.	**-.636**	.206	.335	.084	-.011
I cannot comprehend the robot's inner processes.	**-.619**	.224	.262	-.025	-.025

Table S7. The factor structure matrix after removing items with low factor loadings and items that loaded on multiple factors.

Item	Factor				
	1	2	3	4	5
I feel like the robot's explanations are useful.	**.960**	.128	.021	-.056	-.026
I find the robot's explanations informative.	**.865**	.009	-.012	.007	.021
The robot's explanations make its actions clear to me.	**.847**	-.104	.002	-.090	.001
The robot's explanations for its actions are straightforward.	**.810**	-.142	.006	-.130	-.004
The robot provides information about its actions.	**.804**	-.069	.009	.030	.010
The robot provides clear explanations for its actions.	**.732**	-.098	-.033	.021	.036
The robot ensures that its users are well-informed about its activities.	**.709**	-.017	-.114	.120	-.033
The robot conveys its overall state effectively.	**.650**	-.037	-.091	-.009	.087
The robot explains complex tasks in a way that is easy to understand.	**.604**	.014	-.146	.273	.061
The robot provides detailed explanations of its actions.	**.604**	-.152	.010	.191	-.152
I feel informed about the robot's activities.	**.563**	-.052	-.243	.145	.035
I can predict what the robot will do next.	-.022	**-.873**	.026	.003	.028
It is easy for me to foresee the robot's future actions.	-.037	**-.832**	.024	.036	-.025
I feel confident in predicting the robot's next moves.	.018	**-.792**	-.051	.004	-.060
The robot's behavior is predictable.	.067	**-.782**	.015	.019	-.019
It is easy to anticipate what will follow the robot's behavior.	.063	**-.723**	-.050	-.025	-.052
The robot's next steps are clear to me.	.136	**-.660**	-.071	.089	.114
It is difficult for me to tell what the robot will do next.	.038	**.612**	.300	.182	-.025
The robot provides cues that help predict its next actions.	.229	**-.521**	.032	.180	.214
The robot's actions are obvious.	.281	**-.521**	-.011	-.007	.067
The robot's behavior does not help predict what it will do next.	-.043	**.471**	.263	.135	-.049
The robot's overall functioning is a mystery to me.	-.008	.018	**.765**	.000	.097
It is hard to make sense of the robot's general functioning.	-.084	-.072	**.748**	.068	-.026
I am unsure what the robot does.	-.037	-.011	**.744**	-.077	-.225
It is difficult to get a clear picture of the robot's overall operations.	.012	.055	**.718**	-.117	.088
I cannot comprehend the robot's inner processes.	.032	.065	**.679**	-.040	-.013
I am confused about the robot's general objectives.	-.183	.003	**.671**	.049	.206
I cannot explain the robot's behavior.	.033	.224	**.665**	.154	-.121
It is impossible to know what the robot does.	-.124	.072	**.630**	.080	-.048
It is clear to me what the robot does.	.167	-.139	**-.519**	.260	.100
I have a clear understanding of how the robot operates in general.	.153	-.206	**-.445**	.251	-.223

Table S8. The factor pattern matrix after removing items with low factor loadings and items that loaded on multiple factors.

	I feel informed about the robot's activities.	The robot provides information about its actions.	I find the robot's explanations informative.	The robot's explanations make its actions clear to me.	I feel like the robot's explanations are useful.	The robot provides clear explanations for its actions.	The robot provides detailed explanations of its actions.	The robot's explanations for its actions are straightforward.	The robot explains complex tasks in a way that is easy to understand.	The robot conveys its overall state effectively.	The robot ensures that its users are well-informed about its activities.
I feel informed about the robot's activities.	1.000										
The robot provides information about its actions.	.683	1.000									
I find the robot's explanations informative.	.731	.740	1.000								
The robot's explanations make its actions clear to me.	.713	.759	.753	1.000							
I feel like the robot's explanations are useful.	.659	.689	.750	.739	1.000						
The robot provides clear explanations for its actions.	.663	.743	.743	.757	.680	1.000					
The robot provides detailed explanations of its actions.	.628	.704	.623	.650	.612	.622	1.000				
The robot's explanations for its actions are straightforward.	.692	.732	.738	.793	.728	.698	.641	1.000			
The robot explains complex tasks in a way that is easy to understand.	.654	.686	.705	.655	.647	.685	.651	.639	1.000		
The robot conveys its overall state effectively.	.614	.612	.644	.652	.617	.588	.511	.664	.584	1.000	
The robot ensures that its users are well-informed about its activities.	.722	.715	.718	.747	.677	.705	.640	.680	.691	.623	1.000

Table S9. Inter-Item Correlation Matrix for Factor 1 before removing highly correlated items.

	I feel informed about the robot's activities.	I feel like the robot's explanations are useful.	The robot provides clear explanations for its actions.	The robot provides detailed explanations of its actions.	The robot's explanations for its actions are straightforward.	The robot explains complex tasks in a way that is easy to understand.	The robot conveys its overall state effectively.
I feel informed about the robot's activities.	1.000						
I feel like the robot's explanations are useful.	.659	1.000					
The robot provides clear explanations for its actions.	.663	.680	1.000				
The robot provides detailed explanations of its actions.	.628	.612	.622	1.000			
The robot's explanations for its actions are straightforward.	.692	.728	.698	.641	1.000		
The robot explains complex tasks in a way that is easy to understand.	.654	.647	.685	.651	.639	1.000	
The robot conveys its overall state effectively.	.614	.617	.588	.511	.664	.584	1.000

Table S10. Inter-Item Correlation Matrix for Factor 1 after removing highly correlated items.

	The robot’s actions are obvious.	I can predict what the robot will do next.	The robot’s next steps are clear to me.	I feel confident in predicting the robot’s next moves.	It is easy for me to foresee the robot’s future actions.	It is difficult for me to tell what the robot will do next.	The robot’s behavior is predictable.	The robot provides cues that help predict its next actions.	It is easy to anticipate what will follow the robot’s behavior.	The robot’s behavior does not help predict what it will do next.
The robot’s actions are obvious.	1.000									
I can predict what the robot will do next.	.572	1.000								
The robot’s next steps are clear to me.	.617	.724	1.000							
I feel confident in predicting the robot’s next moves.	.589	.740	.689	1.000						
It is easy for me to foresee the robot’s future actions.	.586	.654	.639	.646	1.000					
It is difficult for me to tell what the robot will do next.	.525	.647	.614	.647	.625	1.000				
The robot’s behavior is predictable.	.646	.668	.690	.671	.642	.594	1.000			
The robot provides cues that help predict its next actions.	.584	.583	.653	.571	.569	.523	.579	1.000		
It is easy to anticipate what will follow the robot’s behavior.	.595	.657	.651	.646	.625	.627	.657	.542	1.000	
The robot’s behavior does not help predict what it will do next.	.501	.557	.545	.542	.522	.613	.542	.479	.536	1.000

Table S11. Inter-Item Correlation Matrix for Factor 2 before removing highly correlated items.

	The robot's actions are obvious.	The robot's next steps are clear to me.	I feel confident in predicting the robot's next moves.	It is easy for me to foresee the robot's future actions.	It is difficult for me to tell what the robot will do next.	The robot's behavior is predictable.	The robot provides cues that help predict its next actions.	It is easy to anticipate what will follow the robot's behavior.	The robot's behavior does not help predict what it will do next.
The robot's actions are obvious.	1.000								
The robot's next steps are clear to me.	.617	1.000							
I feel confident in predicting the robot's next moves.	.589	.689	1.000						
It is easy for me to foresee the robot's future actions.	.586	.639	.646	1.000					
It is difficult for me to tell what the robot will do next.	.525	.614	.647	.625	1.000				
The robot's behavior is predictable.	.646	.690	.671	.642	.594	1.000			
The robot provides cues that help predict its next actions.	.584	.653	.571	.569	.523	.579	1.000		
It is easy to anticipate what will follow the robot's behavior.	.595	.651	.646	.625	.627	.657	.542	1.000	
The robot's behavior does not help predict what it will do next.	.501	.545	.542	.522	.613	.542	.479	.536	1.000

Table S12. Inter-Item Correlation Matrix for Factor 2 after removing highly correlated items.

	I am unsure what the robot does.	It is impossible to know what the robot does.	I cannot explain the robot's behavior.	I cannot comprehend the robot's inner processes.	The robot's overall functioning is a mystery to me.	I am confused about the robot's general objectives.	It is hard to make sense of the robot's general functioning.	It is difficult to get a clear picture of the robot's overall operations.	I have a clear understanding of how the robot operates in general.	It is clear to me what the robot does.
I am unsure what the robot does.	1.000									
It is impossible to know what the robot does.	.587	1.000								
I cannot explain the robot's behavior.	.638	.575	1.000							
I cannot comprehend the robot's inner processes.	.585	.520	.548	1.000						
The robot's overall functioning is a mystery to me.	.548	.614	.640	.542	1.000					
I am confused about the robot's general objectives.	.592	.598	.576	.515	.589	1.000				
It is hard to make sense of the robot's general functioning.	.583	.559	.621	.535	.557	.578	1.000			
It is difficult to get a clear picture of the robot's overall operations.	.552	.564	.577	.561	.622	.592	.542	1.000		
I have a clear understanding of how the robot operates in general.	.539	.497	.490	.508	.557	.626	.520	.570	1.000	
It is clear to me what the robot does.	.683	.597	.563	.518	.578	.586	.560	.586	.647	1.000

Table S13. Inter-Item Correlation Matrix for Factor 3, no items with high correlations were removed.

No	Item	Characteristics	
		Item Difficulty	Item Discrimination
1	The robot's overall functioning is a mystery to me.	.640	.740
2	It is hard to make sense of the robot's general functioning.	.640	.710
3	It is difficult to get a clear picture of the robot's overall operations.	.610	.730
4	I am confused about the robot's general objectives.	.660	.740
5	I am unsure what the robot does.	.660	.750
6	I cannot comprehend the robot's inner processes.	.600	.680
7	I cannot explain the robot's behavior.	.700	.740
8	It is impossible to know what the robot does.	.710	.720
9	It is clear to me what the robot does.	.660	.750
10	I have a clear understanding of how the robot operates in general.	.630	.700
11	I feel like the robot's explanations are useful.	.690	.790
12	The robot explains complex tasks in a way that is easy to understand.	.610	.770
13	The robot provides detailed explanations of its actions.	.580	.720
14	The robot provides clear explanations for its actions.	.660	.790
15	The robot's explanations for its actions are straightforward.	690	.810
16	I feel informed about the robot's activities.	.660	.780
17	The robot conveys its overall state effectively.	.690	.700
18	It is easy for me to foresee the robot's future actions.	.650	.760
19	The robot's behavior is predictable.	.690	.780
20	I feel confident in predicting the robot's next moves.	.650	.780
21	It is easy to anticipate what will follow the robot's behavior.	.660	.760
22	It is difficult for me to tell what the robot will do next.	.660	.740
23	The robot's next steps are clear to me.	.690	.800
24	The robot's actions are obvious.	.680	.720
25	The robot provides cues that help predict its next actions.	.690	.690
26	The robot's behavior does not help predict what it will do next.	.660	.660

Note: Average and range of item discrimination and item difficulty for each factor were as follows:
Factor 1: $M_{Difficulty} = .651$, $\text{range}_{Difficulty} = .600 - .710$; $M_{Discrimination} = .726$, $\text{range}_{Discrimination} = .680 - .750$.
Factor 2: $M_{Difficulty} = .654$, $\text{range}_{Difficulty} = .580 - .690$; $M_{Discrimination} = .766$, $\text{range}_{Discrimination} = .700 - .810$.
Factor 3: $M_{Difficulty} = .670$, $\text{range}_{Difficulty} = .650 - .690$; $M_{Discrimination} = .743$, $\text{range}_{Discrimination} = .660 - .800$.

Table S14. The Item Characteristics demonstrate acceptable Difficulty (.580 - .710) and strong Discrimination ($>$ .600)

Constructs	Cronbach's alpha				Number of items
	Experiment 1	Experiment 2			
		English	German	Italian	
Performance Trust (MDMT)	.910	.936	.905	.872	8
Moral Trust (MDMT)	.940	.962	.921	.912	12
Acceptance (UTAUT)	.950	.963	.939	.952	25
Anxiety (UTAUT)	.730	.827	.807	.761	4
Experience with robots	.850	.876	.793	.824	8

Table S15. Reliabilities of the constructs measured in Experiment 1 and Experiment 2.

Dependent variable: Performance trust (MDMT)							
Parameter	Df	SS	MS	*F* value	*p* value	ηp^2	95 % CI
Explainability condition	1	24.30	24.35	21.26	<.001***	.06	[0.03, 1.00]
Legibility condition	1	15.50	15.48	13.51	<.001***	.04	[0.01, 1.00]
Predictability condition	1	24.60	24.62	21.49	<.001***	.06	[0.03, 1.00]
Prior experience with robots	1	19.50	19.50	17.03	<.001***	.05	[0.02, 1.00]
Explainability*Legibility	1	0.70	0.70	0.61	.437	<.01	[0.00, 1.00]
Explainability*Predictability	1	0.90	0.89	0.78	.378	<.01	[0.00, 1.00]
Legibility*Predictability	1	1.30	1.32	1.16	.283	<.01	[0.00, 1.00]
Explainability*Legibility*Predictability	2	0.00	0.00	0.00	.964	<.01	[0.00, 1.00]
Residuals	313	358.50	1.15				

Table S16. Results of the 3-way ANOVA with the manipulation of the transparency and the language of participants as independent variable, and performance trust as the dependent variable

Dependent variable: Moral trust (MDMT)							
Parameter	Df	SS	MS	*F* value	*p* value	ηp^2	95 % CI
Explainability condition	1	21.10	21.07	13.97	<.001***	.04	[0.01, 1.00]
Legibility condition	1	16.70	16.71	11.08	<.001***	.03	[0.01, 1.00]
Predictability condition	1	0.70	0.74	0.49	.485	<.01	[0.00, 1.00]
Prior experience with robots	1	38.20	38.20	25.34	<.001***	.07	[0.03, 1.00]
Explainability*Legibility	1	0.30	0.31	0.21	.651	<.01	[0.00, 1.00]
Explainability*Predictability	1	2.50	2.50	1.66	.198	.01	[0.00, 1.00]
Legibility*Predictability	1	0.00	0.01	0.01	.928	<.01	[0.00, 1.00]
Explainability*Legibility*Predictability	2	1.20	1.15	0.76	.383	<.01	[0.00, 1.00]
Residuals	313	471.90	1.51				

Table S17. Results of the 3-way ANOVA with the manipulation of explainability, legibility and predictability of the robot's behavior as independent variables, and moral trust as the dependent variable

Dependent variable: Acceptance (UTAUT)							
Parameter	Df	SS	MS	F value	P value	ηp^2	95 % CI
Explainability condition	1	17.27	17.27	19.25	<.001***	.01	[0.00, 1.00]
Legibility condition	1	4.32	4.32	4.81	.029*	<.01	[0.00, 1.00]
Predictability condition	1	5.39	5.39	6.00	.015*	<.01	[0.00, 1.00]
Prior experience with robots	1	18.27	18.27	20.35	<.001***	.01	[0.00, 1.00]
Explainability*Legibility	1	1.44	1.44	1.61	.206	<.01	[0.00, 1.00]
Explainability*Predictability	1	0.06	0.06	0.07	.795	<.01	[0.00, 1.00]
Legibility*Predictability	1	0.00	0.00	0.00	.989	<.01	[0.00, 1.00]
Explainability*Legibility*Predictability	2	0.25	0.25	0.28	.600	<.01	[0.00, 1.00]
Residuals	313	280.90	0.90				

Table S18. Results of the 3-way ANOVA with the manipulation of explainability, legibility and predictability of the robot's behavior as independent variables, and acceptance as the dependent variable

Dependent variable: Anxiety (UTAUT)							
Parameter	Df	SS	MS	*F* value	*p* value	ηp^2	95 % CI
Explainability condition	1	4.90	4.90	3.40	.066	.01	[0.00, 1.00]
Legibility condition	1	2.10	2.09	1.45	.229	<.01	[0.00, 1.00]
Predictability condition	1	0.10	0.07	0.05	.824	<.01	[0.00, 1.00]
Prior experience with robots	1	2.40	2.40	1.67	.197	.01	[0.00, 1.00]
Explainability*Legibility	1	0.70	0.68	0.474	.492	<.01	[0.00, 1.00]
Explainability*Predictability	1	0.70	0.69	0.478	.490	<.01	[0.00, 1.00]
Legibility*Predictability	1	1.10	1.12	0.77	.989	<.01	[0.00, 1.00]
Explainability*Legibility*Predictability	2	0.20	0.21	0.14	.704	<.01	[0.00, 1.00]
Residuals	313	450.40	1.44				

Table S19. Results of the 3-way ANOVA with the manipulation of explainability, legibility and predictability of the robot's behavior as independent variables, and anxiety as the dependent variable

Experiment 2

Language	Condition	No. of Participants	Age		Gender			Experience with robots	
			M	SD	Male	Female	Other	M	SD
English	Condition 1 - **High Transparency**	151	44.13	14.29	71	78	2	1.84	1.11
	Condition 2 - **Low Transparency**	149	42.70	14.57	57	90	2	1.82	1.13
German	Condition 1 - **High Transparency**	144	36.24	11.50	76	64	4	2.00	1.19
	Condition 2 - **Low Transparency**	156	36.44	12.18	86	65	5	1.98	1.11
Italian	Condition 1 - **High Transparency**	150	33.26	10.73	89	57	4	1.97	1.20
	Condition 2 - **Low Transparency**	151	32.98	9.13	73	73	5	1.98	1.23
**Total**		**901**	**37.62**	**12.96**	**452**	**427**	**22**	**1.93**	**1.16**

Table S20. Demographic information across the three languages and two conditions.

Standards	English	German	Italian	Acceptable	Excellent
Minimum fit function chi-square (χ^2)	1854.350	1877.748	1630.673	-	-
Degrees of freedom (df)	299	299	299		
χ^2/df	6.202	6.280	5.454	< 5.00	< 3.00
GFI	0.544	0.535	0.578	> 0.80	> 0.90
RMSEA	0.132	0.133	0.122	< 0.08	< 0.06
AGFI	0.465	0.454	0.505	> 0.80	> 0.90
NFI	0.743	0.743	0.777	> 0.85	> 0.90
CFI	0.774	0.774	0.809	> 0.90	> 0.95
TLI	0.755	0.754	0.793	> 0.90	> 0.95
IFI	0.755	0.775	0.810	> 0.90	> 0.95
SRMR	0.080	0.083	0.074	< 0.08	< 0.05

Table S21. Comparative Fit Indices of a 1-Factor model in English, German, and Italian languages in Experiment 2

Standards	English	German	Italian	Acceptable	Excellent
Minimum fit function chi-square (χ^2)	1492.204	1475.594	1298.833	-	-
Degrees of freedom (df)	298	298	298		
χ^2/df	5.007	4.952	4.359	< 5.00	< 3.00
GFI	0.623	0.626	0.658	> 0.80	> 0.90
RMSEA	0.116	0.115	0.106	< 0.08	< 0.06
AGFI	0.556	0.559	0.597	> 0.80	> 0.90
NFI	0.793	0.798	0.822	> 0.85	> 0.90
CFI	0.827	0.831	0.857	> 0.90	> 0.95
TLI	0.811	0.816	0.844	> 0.90	> 0.95
IFI	0.828	0.832	0.857	> 0.90	> 0.95
SRMR	0.076	0.078	0.066	< 0.08	< 0.05

Table S22. Comparative Fit Indices of a 2-Factor model in English, German, and Italian languages in Experiment 2

Standards	English	German	Italian	Acceptable	Excellent
Minimum fit function chi-square (χ^2)	864.821	1124.839	784.424	-	-
Degrees of freedom (df)	293	293	293		
χ^2/df	2.952	3.839	2.677	< 5.00	< 3.00
GFI	0.815	0.762	0.826	> 0.80	> 0.90
RMSEA	0.081	0.097	0.075	< 0.08	< 0.06
AGFI	0.778	0.714	0.791	> 0.80	> 0.90
NFI	0.880	0.846	0.893	> 0.85	> 0.90
CFI	0.917	0.881	0.930	> 0.90	> 0.95
TLI	0.908	0.868	0.922	> 0.90	> 0.95
IFI	0.917	0.881	0.930	> 0.90	> 0.95
SRMR	0.074	0.076	0.065	< 0.08	< 0.05

Table S23. Comparative Fit Indices of a 4-Factor model in English, German, and Italian languages in Experiment 2

Dependent variable: Performance Trust (MDMT)							
	Df	SS	MS	F value	p value	ηp^2	95 % CI
Transparency condition	1	187.90	187.89	141.46	<.001 ***	.14	[0.10, 1.00]
Language	2	190.40	95.18	71.66	<.001***	.14	[0.10, 1.00]
Transparency condition * Language	2	11.00	5.52	4.15	.016*	.01	[0.00, 1.00]
Residuals	895	1188.8	1.33				

Table S24. Results of the 2-way ANOVA with the manipulation of the transparency and the language of participants as independent variable, and Performance trust as the dependent variable

Dependent variable: Moral Trust (MDMT)							
Parameter	Df	SS	MS	F value	p value	ηp^2	95 % CI
Transparency condition	1	70.40	70.38	43.12	<.001 ***	.05	[0.03, 1.00]
Language	2	490.80	245.41	150.35	<.001***	.25	[0.21, 1.00]
Transparency condition * Language	2	22.90	11.46	7.02	<.001	.02	[0.00, 1.00]
Residuals	895	1460.80	1.63				

Table S25. Results of the 2-way ANOVA with the manipulation of the transparency and the language of participants as independent variable, and Moral trust as the dependent variable

Dependent variable: Acceptance (UTAUT)							
Parameter	Df	SS	MS	F value	p value	ηp^2	95 % CI
Transparency condition	1	59.70	59.73	43.44	<.001 ***	.05	[0.03, 1.00]
Language	2	24.40	12.18	8.86	<.001***	.02	[0.01, 1.00]
Transparency condition * Language	2	1.80	0.90	0.651	.521	<.01	[0.00, 1.00]
Residuals	895	1230.60	1.37				

Table S26. Results of the 2-way ANOVA with the manipulation of the transparency and the language of participants as independent variable, and Acceptance as the dependent variable

Dependent variable: Anxiety (UTAUT)							
	Df	SS	MS	F value	p value	ηp^2	95 % CI
Transparency condition	1	33.80	33.84	20.05	<.001 ***	.02	[0.01, 1.00]
Language	2	12.80	6.39	3.79	<.023*	.01	[0.01, 1.00]
Transparency condition * Language	2	0.30	0.13	0.08	.924	<.01	[0.00, 1.00]
Residuals	895	1510.30	1.69				

Table S27. Results of the 2-way ANOVA with the manipulation of the transparency and the language of participants as independent variable, and Anxiety as the dependent variable
