# Supplementary material for: Measuring transparency in intelligent robots
Source: Sci Rep. 2025 Dec 12;15:43809. doi: 10.1038/s41598-025-29685-w (PMC12705673; doi:10.1038/s41598-025-29685-w)
Supplement: Supplementary file 2 — Supplementary Information 2. [file 41598_2025_29685_MOESM2_ESM.pdf]

Transparency Of RObots Scale (TOROS) - English Version

Georgios Angelopoulos[1,*,+], Dimitri Lacroix[2,,+], Ricarda Wullenkord[2], Alessandra Rossi[1], Silvia Rossi[1], and Friederike Eyssel[2]**

[1]Interdepartmental Center for Advances in Robotic Surgery - ICAROS, University of Naples Federico II, Naples, 80131, Italy
[2]Center for Cognitive Interaction Technology - CITEC, Bielefeld University, Bielefeld, 33619, Germany
[*]georgios.angelopoulos@unina.it
[**]dimitri.lacroix@uni-bielefeld.de
[+]these authors contributed equally to this work

Instructions

Contextualized instructions for participants:

The following statements are about the robot, its behaviors, and its functioning. Please indicate the degree to which you disagree or agree with these statements (from 1 “Strongly disagree” to 7 “Strongly agree”).

Not-contextualized instructions for participants:

Please indicate the degree to which you disagree or agree with the following statements (from 1 “Strongly disagree” to 7 “Strongly agree”).

Instructions for scoring:

By default, this questionnaire is a 7-point Likert scale (labels based on[1,2]).

1	2	3	4	5	6	7
Strongly disagree	Disagree	Somewhat disagree	Neither agree or disagree	Somewhat agree	Agree	Strongly agree

The scale can be converted as a 5-point Likert scale using the following scaling.

1	2	3	4	5
Strongly disagree	Disagree	Neither agree or disagree	Agree	Strongly agree

The authors, however, strongly recommend not to do so, as 7-point Likert scales present the best balance between ease of use, adjustment to memory span, and accuracy[1].

Instructions for administration:

The order of the presented items should be ideally randomized.

Instructions for scoring:

Subscale (dimension) scores are calculated by averaging the ratings of the items of each subscale. A composite score of transparency can be calculated with the average of the three subscales.

The items:

Factors	Items
Illegibility	The robot's overall functioning is a mystery to me.<br>It is hard to make sense of the robot's general functioning.<br>It is difficult to get a clear picture of the robot's overall operations<br>I am confused about the robot's general objectives.<br>I am unsure what the robot does.<br>I cannot comprehend the robot's inner processes.<br>I cannot explain the robot's behavior.<br>It is impossible to know what the robot does.<br>It is clear to me what the robot does. (R)<br>I have a clear understanding of how the robot operates in general. (R)
Explainability	I feel like the robot's explanations are useful.<br>The robot explains complex tasks in a way that is easy to understand.<br>The robot provides detailed explanations of its actions.<br>The robot provides clear explanations for its actions.<br>The robot's explanations for its actions are straightforward.<br>I feel informed about the robot's activities.<br>The robot conveys its overall state effectively.
Predictability	It is easy for me to foresee the robot's future actions.<br>The robot's behavior is predictable.<br>I feel confident in predicting the robot's next moves.<br>It is easy to anticipate what will follow the robot's behavior.<br>It is difficult for me to tell what the robot will do next. (R)<br>The robot's next steps are clear to me.<br>The robot's actions are obvious.<br>The robot provides cues that help predict its next actions.<br>The robot's behavior does not help predict what it will do next. (R)

Note: (R) indicates reverse-coded items that require score inversion before analysis.

References

1. Taherdoost, H. What is the best response scale for survey and questionnaire design; review of different lengths of rating scale/attitude scale/likert scale. *Hamed Taherdoost* 1–10 (2019).

2. Wade, M. V. *et al.* Likert-type scale response anchors. *Clemson international institute for tourism & research development, department parks, recreation tourism management. Clemson Univ.* 4–5 (2006).
