# Supplementary material for: Measuring transparency in intelligent robots
Source: Sci Rep. 2025 Dec 12;15:43809. doi: 10.1038/s41598-025-29685-w (PMC12705673; doi:10.1038/s41598-025-29685-w)
Supplement: Supplementary file 3 — Supplementary Information 3. [file 41598_2025_29685_MOESM3_ESM.pdf]

Transparency Of RObots Scale (TOROS) - German Version

Georgios Angelopoulos[1,*,+], Dimitri Lacroix[2,,+], Ricarda Wullenkord[2], Alessandra Rossi[1], Silvia Rossi[1], and Friederike Eyssel[2]**

[1]Interdepartmental Center for Advances in Robotic Surgery - ICAROS, University of Naples Federico II, Naples, 80131, Italy
[2]Center for Cognitive Interaction Technology - CITEC, Bielefeld University, Bielefeld, 33619, Germany
[*]georgios.angelopoulos@unina.it
[**]dimitri.lacroix@uni-bielefeld.de
[+]these authors contributed equally to this work

Instruktionen

Kontextabhängig:
Die folgenden Aussagen beziehen sich auf den Roboter, seine Verhaltensweisen und seine Funktionsweise. Bitte geben Sie an, inwieweit Sie diesen Aussagen zustimmen oder sie ablehnen (von 1 “lehne stark ab” bis 7 “stimme stark zu”).

Nicht kontextualisiert:
Sie diesen Aussagen zustimmen oder sie ablehnen (von 1 “lehne stark ab” bis 7 “stimme stark zu”).

Hinweis:
Standardmäßig handelt es sich bei diesem Fragebogen um eine 7-Punkte-Likert-Skala[1,2]).

1	2	3	4	5	6	7
lehne stark ab	lehne ab	lehne etwas ab	stimme weder zu noch lehne es ab	stimme eher zu	stimme zu	stimme stark zu

Die Skala kann anhand der folgenden Skalierung in eine 5-Punkte-Likert-Skala umgewandelt werden.

1	2	3	4	5
lehne stark ab	lehne ab	stimme weder zu noch lehne es ab	stimme zu	stimme stark zu

Die Autoren empfehlen jedoch dringend, dies nicht zu tun, da 7-Punkte-Likert-Skalen die beste Balance zwischen Benutzerfreundlichkeit, Anpassung an die Gedächtnisspanne und Genauigkeit bieten[1].

Anweisungen für die Durchführung:
Die Reihenfolge der präsentierten Items sollte idealerweise randomisiert sein.

Hinweise zur Auswertung:
Die Subskalen (Dimensionen) werden durch Mittelung der Bewertungen der Items der einzelnen Subskalen berechnet. Aus dem Durchschnitt der drei Subskalen kann ein zusammengesetzter Wert für die Transparenz berechnet werden.

Die Items:

Faktoren	Die Items
Unschärfe	Die allgemeine Funktionsweise des Roboters ist für mich ein Rätsel.<br>Es ist schwierig, die allgemeine Funktionsweise des Roboters zu verstehen.<br>Es ist schwierig, sich ein klares Bild von der allgemeinen Funktionsweise des Roboters zu machen.<br>Ich bin über die allgemeinen Ziele des Roboters verwirrt.<br>Ich bin unsicher, was der Roboter macht.<br>Ich kann die inneren Vorgänge des Roboters nicht nachvollziehen.<br>Ich kann mir das Verhalten des Roboters nicht erklären.<br>Es ist unmöglich zu wissen, was der Roboter tut.<br>Es ist mir klar, was der Roboter macht. (R)<br>Ich habe eine klare Vorstellung davon, wie der Roboter im Allgemeinen funktioniert. (R)
Erklärbarkeit	Ich habe das Gefühl, dass die Erklärungen des Roboters nützlich sind.<br>Der Roboter erklärt komplexe Aufgaben auf eine leicht verständliche Weise.<br>Der Roboter gibt detaillierte Erklärungen für seine Handlungen ab.<br>Der Roboter liefert klare Erklärungen für seine Handlungen.<br>Die Erklärungen des Roboters für seine Handlungen sind einfach.<br>Ich fühle mich über die Aktivitäten des Roboters informiert.<br>Der Roboter vermittelt seinen allgemeinen Zustand effektiv.
Vorhersehbarkeit	Es fällt mir leicht, die zukünftigen Aktionen des Roboters vorherzusehen.<br>Das Verhalten des Roboters ist vorhersehbar.<br>Ich bin zuversichtlich, dass ich die nächsten Schritte des Roboters vorhersagen kann.<br>Es ist leicht vorauszusehen, was auf das Verhalten des Roboters folgen wird.<br>Es ist schwierig für mich zu sagen, was der Roboter als nächstes tun wird. (R)<br>Die nächsten Schritte des Roboters sind für mich klar.<br>Die Aktionen des Roboters sind offensichtlich.<br>Der Roboter gibt Hinweise, die helfen, seine nächsten Handlungen vorherzusagen.<br>Das Verhalten des Roboters hilft nicht dabei vorherzusagen, was er als nächstes tun wird. (R)

Hinweis: (R) kennzeichnet umgekehrt codierte Items, deren Bewertungen vor der Analyse invertiert werden müssen.

References

1. Taherdoost, H. What Is the Best Response Scale for Survey and Questionnaire Design; Review of Different Lengths of Rating Scale / Attitude Scale / Likert Scale. *Int. J. Acad. Res. Manag.* **8**, 1–10 (2022).
2. Wade, M. V. *et al.* Likert-type scale response anchors. *Clemson international institute for tourism & research development, department parks, recreation tourism management. Clemson Univ.* 4–5 (2006).

Acknowledgements

Die Autoren danken Lena Schubert für ihren Beitrag zur Übersetzung der Skala in die deutsche Sprache.
