# Supplementary material for: Measuring transparency in intelligent robots
Source: Sci Rep. 2025 Dec 12;15:43809. doi: 10.1038/s41598-025-29685-w (PMC12705673; doi:10.1038/s41598-025-29685-w)
Supplement: Supplementary file 4 — Supplementary Information 4. [file 41598_2025_29685_MOESM4_ESM.pdf]

Transparency Of RObots Scale (TOROS) - Italian Version

Georgios Angelopoulos[1,*,+], **Dimitri Lacroix**[2,**,+], **Ricarda Wullenkord**[2], **Alessandra Rossi**[1], **Silvia Rossi**[1], **and Friederike Eyssel**[2]

[1]Interdepartmental Center for Advances in Robotic Surgery - ICAROS, University of Naples Federico II, Naples, 80131, Italy
[2]Center for Cognitive Interaction Technology - CITEC, Bielefeld University, Bielefeld, 33619, Germany
[*]georgios.angelopoulos@unina.it
[**]dimitri.lacroix@uni-bielefeld.de
[+]these authors contributed equally to this work

Istruzioni

Istruzioni contestualizzate per i partecipanti:

Le seguenti affermazioni riguardano i robot, i loro comportamenti e le loro funzionalità. Per piacere indicate il grado con cui siete in disaccordo o in accordo con queste affermazioni (da 1 “Fortemente in disaccordo” a 7 “Fortemente d’accordo”).

Istruzioni non contestualizzate per i partecipanti:

Per piacere indicate il grado con cui siete in disaccordo o in accordo con queste affermazioni (da 1 “Fortemente in disaccordo” a 7 “Fortemente d’accordo”).

Istruzioni per la valutazione:

i base questo questionario è una scala Likert a 7 punte (le etichette sono basate su[1,2]).

1	2	3	4	5	6	7
Fortemente in disaccordo	In disaccordo	Abbastanza in disaccordo	Né d’accordo né in disaccordo	Abbastanza d’accordo	D’accordo	Fortemente d’accordo

Tuttavia la scala qui proposta (scala Likert a 7 punte) può essere convertita in nella seguente versione ridotta (scala Likert a 5 punte).

1	2	3	4	5
Fortemente in disaccordo	In disaccordo	Né d’accordo né in disaccordo	D’accordo	Fortemente d’accordo

Gli autori raccomandano l’utilizzo della versione di scale Likert a 7 punte in quanto rappresentano il miglior bilanciamento tra facilità d’uso, adeguamento alla capacità di associazione, e accuratezza[1].

Istruzioni per la somministrazione:

Gli autori suggeriscono di presentare gli elementi della scala in ordine casuale.

Istruzioni per la valutazione:

I punteggi della Sottoscala (dimensione) sono calcolati facendo una media semplice della valutazione dei singoli elementi di ogni sottoscala. Il punteggio composto della trasparenza può essere calcolato come la media delle tre sottoscale.

Le voci:

Fattori	Le voci
Illeggibilità	Il funzionamento generale del robot è un mistero per me.<br>E' difficile capire il generale funzionamento del robot.<br>E' difficile avere una chiara visione delle operazioni generali del robot.<br>Sono confuso sul obiettivo generale del robot.<br>Non sono sicuro di cosa faccia il robot.<br>Non capisco quali siano i processi interni del robot.<br>Non so spiegare il comportamento del robot.<br>E' impossibile sapere cosa il robot faccia.<br>Mi è chiaro cosa il robot faccia.<br>Ho una chiara comprensione sul come il robot operi in generale. (R)<br>Ho l'impressione che le spiegazioni del robot siano utili. (R)
Spiegabilità	Il robot spiega task complessi in un modo semplice da capire.<br>Il robot da spiegazioni dettagliate delle sue azioni.<br>Il robot da chiare spiegazioni delle sue azioni.<br>Le spiegazioni date dal robot sulle sue azioni sono dirette.<br>Mi sento informato riguardo le attività del robot.<br>Il robot comunica il suo stato generale in maniera effettiva.<br>E' facile per me prevedere le future azioni del robot.
Prevedibilità	Il comportamento del robot è prevedibile.<br>Mi sento sicuro nel predire i movimenti successivi del robot.<br>E' facile anticipare cosa avverrà dal comportamento del robot.<br>E' difficile per me dire cosa il robot farà successivamente. (R)<br>Le prossime azioni del robot sono chiare per me.<br>Le azioni del robot sono scontate.<br>Il robot da indizi che aiutano a predire le sue successive azioni.<br>Il comportamento del robot non aiuta a predire cosa farà successivamente. (R)

Nota: (R) indica voci con codifica inversa, i cui punteggi devono essere invertiti prima dell'analisi.

References

1. Taherdoost, H. What Is the Best Response Scale for Survey and Questionnaire Design; Review of Different Lengths of Rating Scale / Attitude Scale / Likert Scale. *Int. J. Acad. Res. Manag.* **8**, 1–10 (2022).
2. Wade, M. V. *et al.* Likert-type scale response anchors. *Clemson international institute for tourism & research development, department parks, recreation tourism management. Clemson Univ.* 4–5 (2006).

Acknowledgements

Gli autori ringraziano Francesco Vigni per il suo contributo alla traduzione della scala in lingua Italiana.
